# Assessment of secular trends of three major gynecologic cancers burden and attributable risk factors from 1990 to 2019: an age period cohort analysis

**DOI:** 10.1186/s12889-024-18858-3

**Published:** 2024-05-19

**Authors:** Yiran Liu, Wenqi Shi, Sumaira Mubarik, Fang Wang

**Affiliations:** 1 The Second Clinical Medical College of Nanjing Medical University, Nanjing, China; 2 Department of Biostatistics, School of Public Health, Xuzhou Medical University, Xuzhou, China; 3 Department of PharmacoTherapy-Epidemiology and Economics, Groningen Research Institute of Pharmacy, University of Groningen, Groningen, the Netherlands; 4 Key Laboratory of Human Genetics and Environmental Medicine, Xuzhou Medical University, Xuzhou, China

**Keywords:** Global burden of disease, Cervical cancer, Ovarian cancer, Uterine cancer, Age-period-cohort model

## Abstract

**Background:**

This study aims to assess the long-term trends in the burden of three major gynecologic cancers(GCs) stratified by social-demographic status across the world from 1990 to 2019. To assess the trends of risk factor attributed mortality, and to examine the specific effects of age, period, cohort behind them in different regions.

**Methods:**

We extracted data on the mortality, disability-adjusted life years(DALYs), and age-standardized rates(ASRs) of cervical cancer(CC), uterine cancer(UC), and ovarian cancer(OC) related to risks from 1990 to 2019, as GCs burden measures. Age-period-cohort analysis was used to analyze trends in attributable mortality rates.

**Results:**

The number of deaths and DALYs for CC, UC and OC increased since 1990 worldwide, while the ASDRs decreased. Regionally, the ASDR of CC was the highest in low SDI region at 15.05(11.92, 18.46) per 100,000 in 2019, while the ASDRs of UC and OC were highest in high SDI region at 2.52(2.32,2.64), and 5.67(5.16,6.09). The risk of CC death caused by unsafe sex increased with age and then gradually stabilized, with regional differences. The period effect of CC death attributed to smoking showed a downward trend. The cohort effect of UC death attributed to high BMI decreased in each region, especially in the early period in middle, low-middle and low SDI areas.

**Conclusions:**

Global secular trends of attributed mortality for the three GCs and their age, period, and cohort effects may reflect the diagnosis and treatment progress, rapid socioeconomic transitions, concomitant changes in lifestyle and behavioral patterns in different developing regions. Prevention and controllable measures should be carried out according to the epidemic status in different countries, raising awareness of risk factors to reduce future burden.

**Supplementary Information:**

The online version contains supplementary material available at 10.1186/s12889-024-18858-3.

## Background

Cervical cancer (CC), uterine cancer (UC), and ovarian cancer (OC) are the three most common gynecologic cancers (GCs) of the female reproductive system, which are also contributing significantly to female cancer-related deaths and imposing a heavy burden on healthcare [1]. According to GLOBOCAN 2020 database, all these three tumors of the female reproductive system are among the top 10 most common cancer types [2].

Epidemiological data suggests that the prevalence trends of GCs vary across different regions over time [3–5]. CC exhibits an uneven distribution of incidence and mortality in the world, with the highest rates in Eastern and Southern Africa [6]. OC has a poor prognosis as it is often diagnosed at an advanced stage due to its hidden onset, and the lack of effective screening and early diagnostic methods [7]. With the economic transformation and social development, GC has gradually occupied the dominant position in the cause of disease in underdeveloped regions, bringing heavy burden [8]. The United Nations’ Sustainable Development Strategy aims to reduce premature mortality from NCDS by at least 30% by the middle of this century [9]. As GC is an important component of the burden on women, it is crucial to discuss regional disparities and explore its risk factors.

Risk factors for GC generally include behavioral, environmental, genetics, reproductive factors and there are also regional differences. UC and OC are usually associated with increased obesity, and estrogen-related exposures, which seem to be more common in developed countries [3, 10]. CC is associated with chronic sexually transmitted human papillomavirus(HPV) infection, which mainly affects women in less developed regions [11]. However, it is unclear whether this trend has changed in recent years. Zhao et al. studied the risk factors for four types of cancer, but it was conducted on specific populations [12]. Zhou et al. believed that the increase in OC incidence was mainly attributed to population growth and changes in age structure [10]. Therefore, decomposing GC trends from the perspectives of age, period, and cohort can help us to further explore the reasons behind these trends more deeply.

In this study, based on the age-period-cohort(APC) framework, we used data from the global burden of disease study (GBD) 2019 to analyze the distribution and the long-term trends of death, disability-adjusted life years (DALYs), and risk factors for GCs in different regions around the world over the past three decades, and analyze their age, period and cohort effects. The results can provide valuable information for improving the allocation of healthcare resources in different regions, especially for vulnerable populations, and also provide insights for etiological research on GC.

## Methods

### Data source

Annual data on death, DALYs, and their corresponding age-standardized rates (ASRs) and risk factors attributable to GCs (CC, UC and OC) for women were obtained from the GBD 2019 database (https://ghdx.healthdata.org/). The GBD 2019 study provides annual estimates of incidence, prevalence, mortality, years of life lost, years lived with disability, and DALYs for 369 diseases and injuries from 1990 to 2019. General methodology details were available on the website help page and in previous publications [13].

### Definitions

The causes of death for CC, UC and OC were identified according to the 10th revision of the international classification of diseases. The variables included death cases, DALYs numbers, and their corresponding crude and ASRs at the global and regional levels. Disability-adjusted life year (DALY), as a comprehensive indicator that reflects the health status of a certain population across time and periods, is equal to the sum of years of life lost (YLL) and years lived with disability (YLD) [14]. One DALY is equivalent to one year of healthy life lost. The number of YLL due to GCs was obtained by subtracting the age at death from the life expectancy for a person of that age. YLD was derived by multiplying the prevalence of each sequela of GCs by its disability weight. Age-standardized rates of mortality and DALYs were based on the GBD 2019 global age-standard population.

These data were extracted and stratified by age (15–19, every 5-year age group up to 95 years), calendar year (1990–2019), country and territory. Geographically, all 204 countries and territories were divided into five quintiles according to socio-demographic index (SDI) [15] in the GBD study, which we also used as the basis for regional grouping. SDI is used to measure the social development level, it is a composite indicator of per capita income, average education for individuals aged 15 and older, and the total fertility rate for females under 25, which ranges from 0 to 1 [15]. Based on SDI values, countries and territories are classified into 5 quintiles: high (countries with SDI levels between 0.805 and 1.000, such as USA, England, and Germany); high-middle (0.689 to 0.805, such as Malaysia, Spain, and Portugal); middle (0.608 to 0.689, such as China, Mexico, and Brazil); low-middle (0.455 to 0.608, such as Bangladesh, India), and low (less than 0.455, such as Central African Republic and Ethiopia) SDI regions.

### Attributable risk factors to GCs

In GBD 2019, risk factors were organized into five hierarchical levels, level 0 reports estimate for all risk factors combined, level 1 includes three risk categories (behavioral, environmental or occupational, and metabolic risks), this hierarchical structure continues, with each subsequent level (level 2–4) containing more detailed risk factors in the broader categories nested within it [16]. Unsafe sex, smoking, high body-mass index (BMI), high fasting plasma glucose (FPG), and occupational asbestos exposure (OAE) are the five important risk variables that will be concerned in this study.

The attributable burden caused by risk factors was calculated using the comparative risk assessment framework (CRA) in GBD 2019, which was based on the statement that the amount of disease burden can be reduced by reducing exposure to a specific risk factor to the theoretical minimum risk exposure level [16]. According to CRA, assuming that exposure levels of other risk factors remain unchanged, the theoretical minimum risk exposure distributions of selected risks were compared with the exposure distributions of a certain population, then the population attributable fractions (PAF) of each risk was estimated [17]. The burden of disease attributable to a selected risk factor could be obtained by multiplying the PAF by the corresponding disease burden indicator. GCs deaths attributable to selected factors were calculated by multiplying the PAFs and total disease-specific deaths.

### Statistical analysis

To characterize the burden of CC, UC, and OC, we first employed a descriptive analysis. The number and ASRs of deaths and DALYs of these three GCs by SDI region in 1990 and 2019 were reported, and the annual rates of change were also reported to reflect the changes in the ASRs of GCs over the past 30 years.

We used the age standardized rates (ASRs) and estimated annual rate of change to quantify the trends of disease burden. When comparing several different populations or the same population with different age structures, ASRs were required. The formula calculation ASR is as follows:

$$ASR = \frac{{\sum _{i = 1}^A{a_i}{w_i}}}{{\bar \sum _{i = 1}^A{w_i}}}$$

Where *a*_*i*_ is the age-specific rate of age group *i*, and *w*_*i*_ is the number of persons in the same age group as the reference standard population. The ASRs were based on the GBD 2019 global standard population. The annual rates of change from 1990 to 2019 were directly extracted from Global Health Data Exchange (GHDx).

As mortality and DALYs represented not only the risk of death experienced by the population in a given year, but also the cumulative health risk since birth. We used the APC framework to assess the three effects of attributable mortality from three GCs among women globally and in different regions. The APC model was designed to evaluate the contribution of age, period, and cohort effects to outcomes [18, 19]. Due to the collinearity, the model had the identification problem. Therefore, we adopted an approach based on the intrinsic estimator (IE) algorithm [18], which solved the APC model from the point view of statistical methods, choosing a solution with the smallest sum of squares of parameters among all possible solutions [19]. Imposes a constraint on parameter estimation, the model based on IE was expressed as: $Y=log\left(M\right)=\mu +\alpha {age}_{i}+\beta {period}_{j}+\gamma {cohort}_{k}+\epsilon$. *M* is defined as the mortality rates. *α* refers to the age effect, the risk of death in a particular age group; *β* is the period effect, which is mortality risk of the population in a given period; *γ* is the cohort effect, the risk of death for all people in the same birth cohort. *μ* is the intercept and *ε* is defined as the random error. The degree of model fitting was evaluated by deviance, Akaike Information Criterion (AIC), and Bayesian Information Criterion (BIC). The standard error (SE) coefficient and risk ratios were calculated. The above statistical description and analyses were performed using the R program (Version 4.1.2, R core team). Results with *P* < 0.05 were considered statistically significant.

## Results

### Global burden of cervical, ovarian, and uterine cancer

In 2019, cervical cancer, ovarian cancer, and uterine cancer caused 280,479, 198,412, and 91,641 deaths globally, with age-standardized death rates of 6.51, 4.56 and 2.09 per 100,000 people respectively (Table 1). From 1990 to 2019, ASDR declined for GCs in most regions except OC. In contrast to areas with high SDI, ASDR of OC increased in middle, low-middle and low SDI quintiles (annual rates of change were 0.50,0.75, and 0.64 respectively).

The DALYs caused by CC was 8955,012.78 person-years in 2019, followed by OC(5359,736.70) and UC(2329,073.70) (Supplementary Table S1). The age-standardized DALY rates decreased globally and in all SDI quintiles from 1990 to 2019, with the largest reduction of -37.36% for CC in high SDI region. The age-standardized DALY rates of OC increased in all areas excepted high and high-middle SDI regions.

**Table 1:** The death cases and age-standardized death rate (ASDR) of cervical, ovarian, and uterine cancer in 1990 and 2019, and its temporal trends from 1990 to 2019

Types	Area	1990			2019		1990–2019
		Death cases	ASDR per 100,000		Death cases	ASDR per 100,000	Annual rate of change
		No.×10^3^(95% UI)	No. (95% UI)		No.×10^3^(95% UI)	No. (95% UI)	No. (95% UI)
**Cervical cancer**							
Overall		184.53(164.84, 218.94)	8.48(7.59, 10.07)		280.48(238.86, 313.93)	6.51(5.55, 7.29)	-0.23(-0.35, -0.12)
SDI							
	High SDI	25.22(23.28, 26.19)	4.56(4.22, 4.71)		26.17(22.82, 28.15)	2.90(2.60, 3.10)	-0.36(-0.40, -0.33)
	High-middle SDI	41.35(38.69, 48.40)	6.95(6.50, 8.13)		51.77(41.66, 57.87)	4.89(3.92, 5.47)	-0.30(-0.46, -0.19)
	Middle SDI	52.53(46.63, 65.12)	9.32(8.31, 11.54)		90.10(71.33, 103.2)	6.78(5.4, 7.76)	-0.27(-0.44, -0.13)
	Low-middle SDI	39.21(32.46, 50.05)	11.71(9.73,15.05)		66.68(57.27, 81.24)	8.85(7.62, 10.83)	-0.24(-0.35, -0.08)
	Low SDI	26.08(20.23, 32.11)	19.18(15, 23.66)		45.54(35.80, 56.26)	15.05(11.92,18.46)	-0.22(-0.34, -0.02)
**Uterine cancer**							
Overall		56.13(51.10, 60.20)	2.67(2.44, 2.86)		91.64(82.39, 101.50)	2.09(1.88, 2.32)	-0.22(-0.27, -0.15)
SDI							
	High SDI	16.88(15.87, 17.41)	2.73(2.57, 2.81)		26.63(24.00, 28.14)	2.52(2.32, 2.64)	-0.08(-0.11, -0.04)
	High-middle SDI	19.52(18.33, 20.67)	3.23(3.03, 3.42)		26.43(23.96, 28.83)	2.33(2.12, 2.55)	-0.28(-0.33, -0.21)
	Middle SDI	11.81(9.28, 13.51)	2.22(1.77, 2.52)		20.95(17.53, 24.33)	1.61(1.36, 1.87)	-0.27(-0.37, -0.12)
	Low-middle SDI	5.57(4.59, 6.75)	1.93(1.61, 2.36)		12.25(10.43, 15.28)	1.75(1.49, 2.21)	-0.10(-0.22, 0.06)
	Low SDI	2.32(1.84, 2.93)	2.08(1.64, 2.65)		5.30(4.32, 6.64)	2.10(1.72, 2.63)	0.01(-0.15, 0.24)
**Ovarian cancer**							
Overall		97.36(89.70, 109.76)	4.59(4.24, 5.16)		198.41(175.36, 217.66)	4.56(4.03, 5.00)	-0.01(-0.14,0.10)
SDI							
	High SDI	43.46(39.18, 45.02)	7.46(6.74, 7.71)		56.64(50.39, 61.32)	5.67(5.16, 6.09)	-0.24(-0.30, -0.12)
	High-middle SDI	29.78(27.76, 31.76)	4.97(4.63, 5.3)		51.97(45.00, 57.25)	4.75(4.11, 5.24)	-0.04(-0.16, 0.06)
	Middle SDI	13.71(12.09, 17.15)	2.44(2.16, 3.05)		48.49(39.89, 56.53)	3.66(3.01, 4.26)	0.50(0.09, 0.79)
	Low-middle SDI	7.31(5.76, 11.58)	2.33(1.84, 3.60)		29.87(24.42, 37.62)	4.09(3.36, 5.15)	0.75(0.10, 1.34)
	Low SDI	3.07(2.12, 6.17)	2.45(1.71, 4.72)		11.35(9.55, 13.93)	4.01(3.38, 4.88)	0.64(-0.02, 1.34)

### Temporal trends in the burden of GCs

**Fig. 1:**
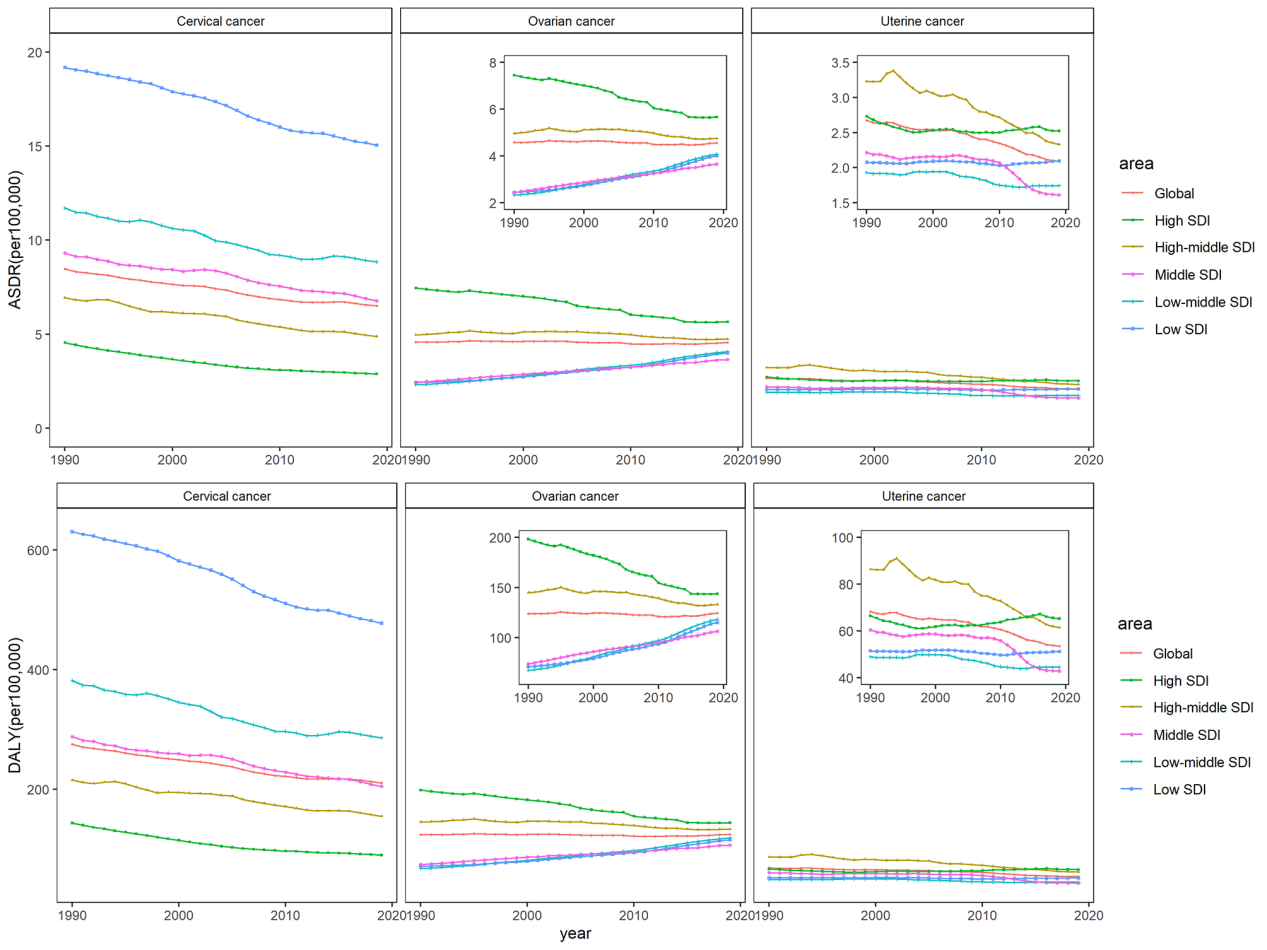
Trends of the age-standardized rate of death (ASDR) and DALYs per 100,000 population for cervical, ovarian, and uterine cancer from 1990 to 2019, globally, and in different social-demographic index regions

Figure 1 shows the yearly ASRs of deaths and DALYs due to CC, UC, and OC globally and in all SDI quintiles from 1990 to 2019. There were significant regional differences in the death trends of the three cancers. The ASDR of CC decreased in all SDI regions over the three decades, and high SDI area remained the lowest level in all regions. For OC, the ASDR was the highest in high SDI area in 1990, with the largest decrease, but the burden in high SDI area remained the highest. By 2019, the disparity of OC levels among different SDI regions had narrowly compared with that in 1990. The ASDR of UC did not change much during the past 30 years, with a slight increase only in low SDI regions. Among all regions, the level of ASDR in high SDI region increased slightly after a decline, making it the first-ranked in 2019 compared to the second-ranked in 1990. The age-standardized DALY rate showed a similar trend as ASDRs.

### Risk factors attributable to the death of GCs

**Fig. 2:**
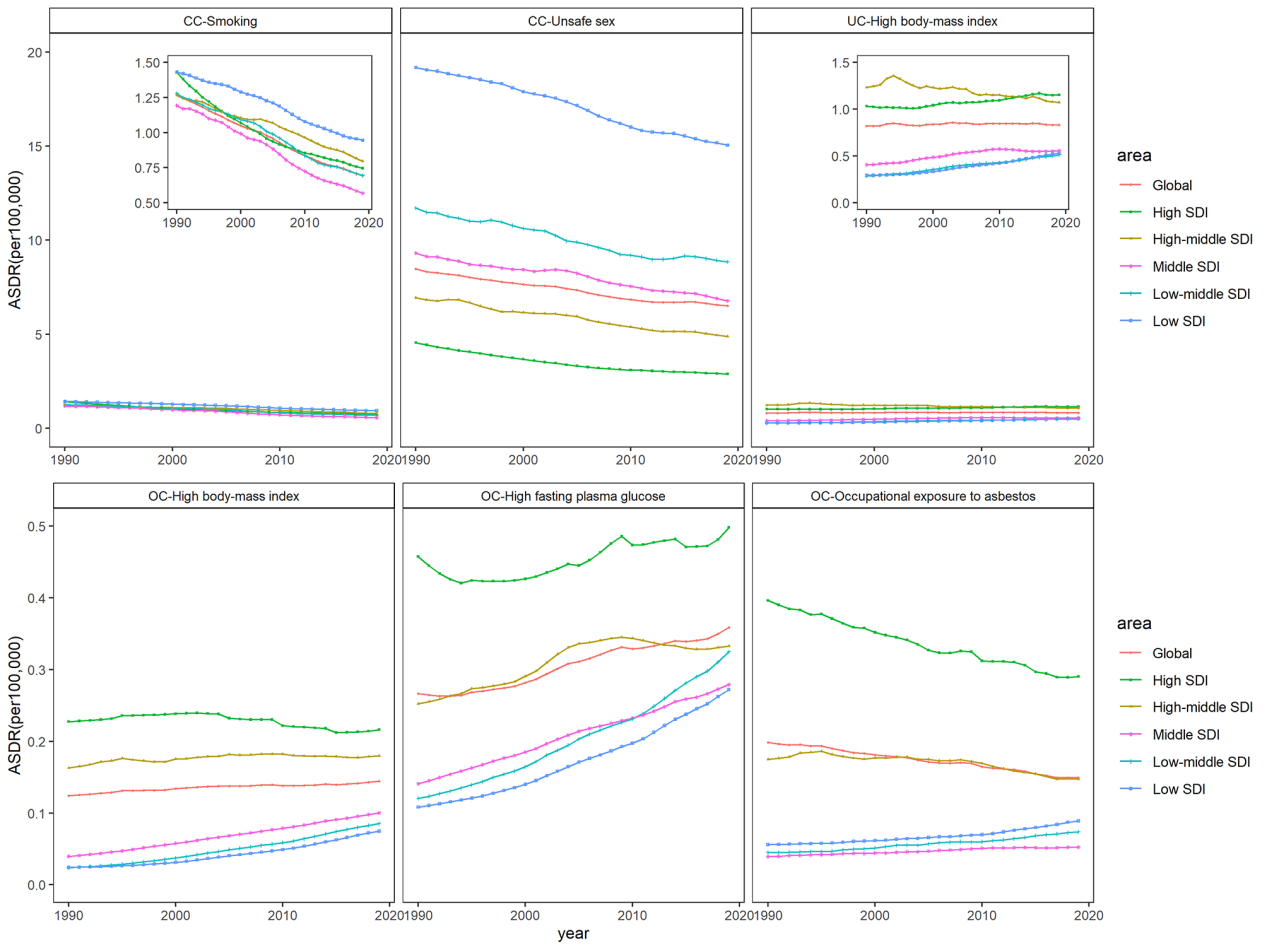
The age-standardized rates of death attributable to related-risk factors for cervical, ovarian, and uterine cancer, globally and regionally, from 1990 to 2019

Globally, unsafe sex behavior was the main risk factor for CC, followed by smoking (Fig. 2). From 1990 to 2019, the ASDR attributed to unsafe sex for CC had shown a consistent decline across the different regions. Smoking contributed relatively little to the burden of CC deaths and has also exhibited a decreasing trend in recent years. In 1990, the ASDR attributed to smoking for CC was similar in low SDI region (1.431 per 100,000 person) and high SDI region (1.430 per 100,000 person). However, during the 30 years, the high SDI regions experienced a greater decline compared to low SDI regions. By 2019, the low SDI region still ranked first among all regions in terms of the attributable mortality of CC.

Figure 2. The age-standardized rates of death attributable to related-risk factors for cervical, ovarian, and uterine cancer, globally and regionally, from 1990 to 2019.

High BMI was the risk factor for UC, with a stable global trend. The highest level of ASDR was found in high SDI region. For OC, high BMI, high FPG, and OAE were the three risk factors, and the ASDR attributed to these factors showed large fluctuations. The attributed ASDR in high SDI region was at a high level among all regions. In middle, low-middle, and low SDI regions, although the attributable burden of OC was relatively low, it showed an increasing trend during the 30 years, especially for high FPG, as shown in Fig. 2.

### Age, period and cohort effects by SDI regions and major factors

**Fig. 3:**
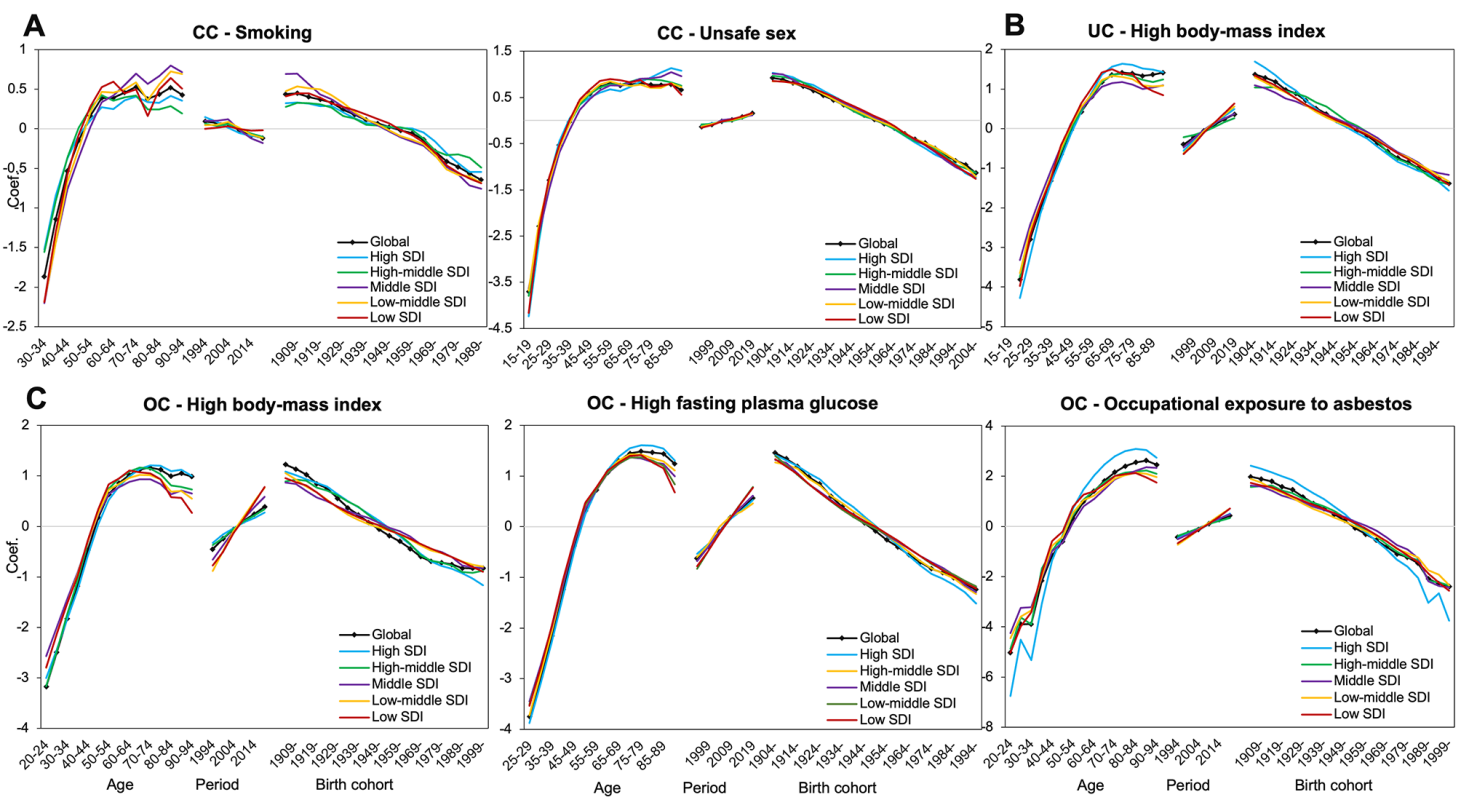
Age-period-cohort related trends in mortality for three cancers from 1990 to 2019, by global and SDI quintiles attributable to risk factors

Figure 3 shows the age, period, and cohort effects of three GCs caused by different risk factors in various SDI regions. For CC, the age and cohort effects of unsafe sex and smoking on ASDR were similar globally, and the segregation trends varied slightly among different SDI regions. As shown in Fig. 3, the risk of death from CC due to unsafe sex increased with age, and gradually stabilized after reaching the 50–54 age group, with regional differences then emerged. In high SDI region, the age effect became statistically significant after 65 years, and reached the highest in the 85–89 age group, with RR and 95%CI of 3.10(1.74, 5.54). The risk of CC death in 85–89 age group was about 4.84 and 1.49 times higher than that of the 30–34 and 65–69 age groups (Table S2). Globally, the period effect increased with years, and the RR(95%CI) was 1.17(1.08,1.26) in 2019. While the period effect was less pronounced in regions with high SDI, and was only significant in low SDI region. The cohort effect of CC mortality showed a downward trend. Later cohorts experienced lower RRs than previous birth cohorts. For CC mortality attributed to smoking, the period effect showed a decreasing trend with fluctuation (Table S3).

Figure 3 Age-period-cohort related trends in mortality for three cancers from 1990 to 2019, by global and SDI quintiles attributable to risk factors.

The death risk of UC attributed to high BMI increased with age, and declined steadily after the age group of 70–74 (4.10[2.22,7.59]) until the 90–94 age group, and showed regional differences. The risk of UC death peaked in the earlier age group (65–69) in the low and low-middle SDI regions (4.47[2.65,7.54], and 3.80[2.52,5.74], respectively) (Table S4). For the period effect, the area with low SDI had the fastest growth rate, and the RR value in 2019 was 3.55 times that in 1990. The cohort effect showed that the effect had gradually decreased in all regions since 1904, but the reduction of birth cohort effect was only significant in the early period in middle, low-middle and low SDI areas. For UC death risk due to high BMI, the RR (95%CI) of death in the 1939–1943 birth cohort was 1.42 (1.02, 1.98), representing a 52% reduction compared with the earliest birth cohort. For the estimated RRs of OC deaths attributed to high BMI and high FPG, the age, period, cohort effects were statistically significant, but the estimated risks of OC mortality attributed to OAE were not significant, as shown in Tables S5 to S7.

## Discussion

This study is a comprehensive analysis into the global and regional CC, UC, and OC burdens under the APC framework. And it also examined the long-term trends of cancer mortality attributed to risk factors in the world and regions, while decomposed their age-, period-, and cohort-specific effects. Overall, the ASDRs and DALYs for CC and UC generally showed a downward trend. The ASDRs and DALY rates for OC showed the declining trend in high SDI region, and an upward trend in middle, low-middle, and low SDI regions, the age trends also differed in different regions, which further suggested that the significant effects of age, period and cohort on the mortality trends of CC, UC and OC caused by different risk factors should be discussed in different regions.

### Cervical cancer

The burden of CC in women as the death number and DALY rate exhibited an overall increasing trend, making it an important disease affecting health. However, the ASDR and AS-DALY rates showed a decreasing trend, suggesting that population aging may exacerbate the death risk and disease burden of CC. Our regional analysis that followed revealed that although the ASDR for CC has decreased in all areas in recent years, regions with high SDI levels have experienced greater decrease, which benefited more than those with lower SDI levels. It suggests that the geographic factor and its socioeconomic correlates are the key stratification parameter [20]. Singh et al. suggested that there are global inequalities in CC mortality due to human development, social inequalities, and differences in living standards [21]. For example, as reported by Jiang D et al. the ASDR of CC in China was lower than that in most developing countries, but higher than that of most developed countries [22]. The 5-year relative survival rate of CC patients in Uganda was 17.7%, much lower than that of black American patients (63.9%) [23]. Women in urban areas were more likely to be tested for CC and have better outcomes than those in rural areas [24]. Research had predicted that if local disparities in hysterectomy incidence rates among high-risk women remain unchanged by 2035, CC rates among black women in older age groups will be significantly higher than those among white women [25].

We found that the increase of CC death cases was relatively high in middle, low-middle and low SDI regions. Although the ASDRs decreased, the rate remained high in low SDI area. The prevalence of CC varied greatly among different regions [26]. Studies had reported that CC was the leading cause of premature death in more than 20 countries worldwide [27], most of which were located in underdeveloped regions such as sub-Saharan Africa, South America, and Southeast Asia, with over 80% of the burden occurring in developing countries [2, 8, 28]. Multiple factors may be associated with CC health disparities, and leading to marked regional differences. We therefore conducted attribution burden analysis for different regions based on the two risk factors included in GBD of CC, and separated the effects.

The age effect reflects the accumulation of various risk factors within the body. After adjusting for period deviations, the results showed that the attributed risk of CC deaths among women in the same birth cohort increased steadily with age, suggesting that women over 50 still faced significant risk of death compared to the younger population. We found that the death risk of CC attributable to smoking increased sharply in women aged 30 and above, and the effect differed among different SDI regions. The attributable risk of death due to unsafe sex started to rapidly increase from 15 years old, and the risk of death was highest among older women in high SDI region. The mortality of CC was positively associated with human papillomavirus, human immunodeficiency virus infection and negatively associated with CC screening coverage [29]. CC most commonly develops in women between 30 and 40 years of age, the recommended age to stop CC screening generally varies between age 50–70 years worldwide [30]. However there is a second incident cases after 70 years of age [31], people tend to reduce the frequency of CC screening or choose not to get screened as they get older. On the other hand, this may also be related to factors such as open-mindedness, early sexual debut and excessive number of sexual partners leading to increased HPV exposure [4].

For the period effect, the death risk of CC attributed to smoking and unsafe sex had different performance. It can be found that the risk was relatively lower in high SDI region, which may in line with the development of medical technology in developed countries, the improvement of screening level leading to early detection of infection, as well as the implementation of tobacco control measures [32]. In contrast, in lower SDI regions with limited healthcare resources and relatively backward screening methods, led to poorer performance [33]. It has been proposed that regions with low SDI often have higher burden of CC, such as Brazil, where CC mortality is estimated to be about twice that of developed countries [34]. With the economic development, the local government launched various action plans, such as to expanding the scope of primary health care, improving screening coverage and vaccination rates, but from the perspective of the urban development process, the process may not be enough to produce a clear and visible positive impact for low SDI areas. For smoking, there was a small downward trend in the period effect in areas with low SDI, which although not statistically significant, may also be able to explain the positive effect of local economic development to some extent. For unsafe sex, it seems that the rapid economic development and people’s increased acceptance of so-called new ideas, had led to changes in their sexual behavior, and increased chance of HPV infection. Studies have reported higher risk of HPV infection among women in sub-Saharan Africa, such as first sexual intercourse and pregnancy at an early age, insufficient condom uses, which increase the risk of HPV infection. In many countries HPV vaccines have been included in routine vaccination programs [35, 36]. For example, in the European region, the target vaccination rate for 15-year-old girls with complete HPV vaccination by 2030 is 90% [35]. In China, the CC vaccine has been approved and used since 2016, reducing the CC incidence caused by HPV infection [37]. However, less than half of low-income countries have implemented national-level vaccination programs for girls, and the vaccine coverage rate is not ideal, CC remains a major public health issue in many countries [38].

### Ovarian cancer

Zhang et al. [3] reported that there were global differences in the incidence patterns of OC, with an increased risk in birth cohorts in Asia, Europe, Central and South America, suggesting a possible association with the prevalence of risk factors such as obesity and smoking, but it did not consider the risk of OC death. Risk factors for OC include breastfeeding, infertility, hormone therapy, and obesity, however, many known risk factors cannot be changed [7, 39]. Therefore, we analyzed the attributable death risk of OC with high BMI, high FPG and OAE risk factors from the perspective of modifiable risk factors.

In general, the age effect of attributed death risk for OC showed a trend of initial increase and subsequent decrease in all regions, with the highest risk occurring between 75 and 85 years of age. Areas with high SDI level had a relatively higher risk among the elderly population. The aging population, along with increased underlying diseases and declined physical fitness among the elderly, will increase the risk of death from OC [40]. We believe that this can be explained similarly to the risk effects of CC.

Period effects reflect the risk caused by changes in the social environment during a certain period. We found that the period effects in all regions have been increasing since 1990, with the changes of social environment, especially in low SDI region. Changes in dietary structure and increased diagnostic capabilities using CT, ultrasound, MRI, and other technologies may lead to higher detection rates of OC [41]. For the birth cohort effect of OC, we found that the later the birth cohort, the lower the risk of death. The overall cohort effect of OC death risk attributed to the three risk factors showed a decreasing trend.

High FPG is one of the important factors for OC. A systematic review of 12 cohort studies suggested that diabetes is associated with higher all-cause and cancer-specific mortality in women with OC [42]. High glucose provides energy to both tumor cells and normal cells simultaneously, diabetes can lead to the occurrence and adverse outcomes of cancer through various pathways, such as programed cell death regulation [10, 43]. OC mortality attributed to high BMI was high and relatively stable in high SDI region, but it has been increasing in middle, low-middle, and low SDI regions over the past 30 years. This may be closely related to socioeconomic development and its uneven distribution. As economic growth has changed the dietary habits of people in low and middle SDI countries, red and processed meat has become more affordable and accessible, the prevalence of obesity or overweight has risen sharply [44, 45]. On the other hand, economic growth had gradually shifted the epidemic pattern of diseases in low- and middle-income countries from infectious diseases, maternal and neonatal diseases, nutritional diseases to non-communicable chronic diseases, with an increased risk of cancer. In addition, improvements in testing with economic growth have made OC easier to detect, but limited technology improvements in low- and middle-income countries may have allowed patients to be first diagnosed at a later stage, resulting in poorer survival, which may also have contributed. Period and cohort effects also indicated a higher risk in low SDI region compared to the reference year. The increase in obesity rate may partially explain the period risk attributed to high BMI attributed OC deaths. With factors such as declining fertility rates, later marriages, and increased unhealthy lifestyles, the burden of OC may rapidly increase in the future. There were significant differences among birth cohorts. The lower mortality risk in the later birth cohort may be attributed to the improvements of living environment and medical conditions in the later birth cohort throughout the life course, which makes it easier for individuals to survive for a longer time and have a lower risk of death.

Currently, OC deaths attributed to OAE still pose a significant burden. Although our results did not show significant age, period and cohort effects, the trends should not be ignored. The risk of death from OC was highest in high SDI regions in the early stage, and this effect has been decreasing in the past 30 years, and reaching its lowest point in 2019. This may be attributed to restrictions on asbestos use and reduced asbestos exposure in some countries. However, high SDI regions still have the highest levels, indicating that governments in these regions should continue their efforts to further restrict the use of asbestos. In addition, the gradually increasing attributable mortality of OC in low and low-middle SDI areas should also be of concern. Conducting occupational disease screening and physical examinations in specific populations will also help to improve the early detection rate and thereby reduce the risk of disease-related deaths [10].

### Uterine cancer

The mortality and DALY rate of UC were relatively lower compared to the other two GCs, making it easy for managers to overlook in disease prevention and treatment work. However, we found that the UC burden presented a higher burden in regions with relatively high SDI level. Barbados in Latin America and the Caribbean, had the highest mortality among women, confirming the high burden in the high-middle SDI region. Even worse, although the number of death cases from UC was relatively low in the low and middle- low SDI areas, the increase in the number of deaths exceeded 100% in both areas. Studies suggests that the ASIR of UC will increase over the next 25 years, also indicating that if corresponding prevention strategies are not taken, the death risk among UC patients will continue to rise [46].

High BMI and obesity are important risk factors for the occurrence and mortality of UC, and the lipid metabolism abnormalities that obesity may bring are also closely related to the occurrence of UC [47, 48]. Compared with UC patients with normal BMI, those with high BMI have poorer clinical outcomes and higher mortality rates. We found that the age effect of UC death risk attributed to high BMI increases with age, and decreases after the 70–74 age group, except in low SDI region, where it peaks one age group earlier.

The aging population and population growth have contributed to the increase in the burden of UC deaths attributed to high BMI over the past three decades. The period effects showed increasing trends in all regions, which may be related to external factors such as socioeconomic level, lifestyle, and medical technology level. With social development, the consumption of unhealthy foods such as high fat and calories has increased significantly, leading to an increase in the obesity rate, which further increases the adverse outcome of UC [49]. The improvement of disease screening levels will also detect more UC patients and report more attributed death cases.

The cohort effect showed that the different exposure levels in different birth cohorts have led to changes in the attributable UC mortality. The cohort effect gradually decreased since the earliest birth cohort. The younger the birth cohort, the higher awareness of health and disease prevention among young people. And they will consciously adopt scientific lifestyles and diet style to control weight. In addition, dietary factors can also affect ovarian lifespan and hormone levels, affecting the age of menopause, which also may reduce the risk of adverse outcomes for UC patients [47].

Studies have reported that the public generally believes that obesity can lead to hypertension, cardiovascular diseases, etc., but little is known about the relationship between obesity and UC [50, 51]. Therefore, it may be necessary to strengthen health education for people in various regions, carry out more comprehensive education on the risk of obesity, improve the population’s awareness of risk factors for UC, and carry out targeted public health interventions to reduce the overall prevalence of obesity in the population may be necessary.

## Conclusions

The burden of GCs was increasing worldwide, with regional differences. There were age, period and cohort effects in the trends of attributed mortality for three GCs. Cervical cancer had the heaviest burden, particularly in low SDI region. Unsafe sex was still the largest influencing factor for CC. Ovarian and uterine cancers were more common in areas with higher SDI. High FPG and BMI were most important risks for death in OC and UC patients, respectively. Although the early burden of OC and UC was relatively low, the increasing trend should not be ignored due to population aging and the prevalence of risk factors in different regions. The burden of UC and OC attributed to risks continues to rise in low SDI area, and without strong intervention, the burden will further increase, bringing a heavy burden in the future. These findings on difference in GCs burden across regions may help resource-limited countries consider how to allocate their healthcare resources.

### Electronic supplementary material

Below is the link to the electronic supplementary material.

Supplementary Material 1

## Data Availability

The datasets generated and analysed during the current study are available at the Global Health Data Exchange website: http://ghdx.healthdata.org/gbd-results-tool.

## Abbreviations

AIC: Akaike Information Criterion
ASR: Age-standardized rate
BIC: Bayesian Information Criterion
BMI: Body-mass index
CC: Cervical cancer
CRA: Comparative risk assessment
DALYs: Disability-adjusted life years
FPG: Fasting plasma glucose
GCs: Gynecologic cancers
OAE: Occupational asbestos exposure
OC: Ovarian cancer
PAF: Population attributable fraction
SDI: Socio-demographic index
SE: Standard error
UC: Uterine cancer
