# Supplementary material for: Assessment of secular trends of three major gynecologic cancers burden and attributable risk factors from 1990 to 2019: an age period cohort analysis
Source: BMC Public Health. 2024 May 19;24:1349. doi: 10.1186/s12889-024-18858-3 (PMC11103856; doi:10.1186/s12889-024-18858-3)
Supplement: Supplementary file 1 — Supplementary Material 1 [file 12889_2024_18858_MOESM1_ESM.docx]

**Table S1.** The DALYs and related age-standardized rate (ASR) of cervical, ovarian, and uterine cancer in 1990 and 2019, and its temporal trends from 1990 to 2019.

| **Types** | **Area** | **1990** | |  | **2019** | | **1990-2019** |
| --- | --- | --- | --- | --- | --- | --- | --- |
|  |  | **all ages** | **ASR per 100,000** |  | **all ages** | **ASR per 100,000** | **Annual rate of change** |
|  |  | **No.×10^3^(95% UI)** | **No. (95% UI)** |  | **No.×10^3^(95% UI)** | **No. (95% UI)** | **No. (95% UI)** |
| **Cervical cancer** | |  |  |  |  |  |  |
| Overall | | 6176.25(5437.67,7316.93) | 275.05(242.75,326.15) |  | 8955.01(7547.73,9978.46) | 210.64(177.67,234.85) | -0.23(-0.35, -0.12) |
| SDI regions | |  |  |  |  |  |  |
|  | High SDI | 725.85(665.20,752.59) | 143.23(130.30,148.45) |  | 672.11(608.75,722.00) | 89.72(81.88,95.85) | -0.37(-0.40, -0.34) |
|  | High-middle SDI | 1274.57(1193.74,1497.01) | 215.21(201.46,252.85) |  | 1543.70(1236,1729.87) | 154.69(124.02,173.51) | -0.28(-0.46, -0.17) |
|  | Middle SDI | 1790.63(1588.48,2223.17) | 287.82(255.02,356.33) |  | 2817.25(2223.19,3217.72) | 204.60(161.92,233.49) | -0.29(-0.46, -0.15) |
|  | Low-middle SDI | 1419.29(1160.75,1789.13) | 381.90(315.26,485.47) |  | 2282.24(1948.33,2722.93) | 285.64(244.64,342.16) | -0.25(-0.36, -0.08) |
|  | Low SDI | 961.20(732.59,1179.64) | 630.59(487.61,777.41) |  | 1632.49(1271.61,2044.29) | 477.53(374.33,591.38) | -0.24(-0.36, -0.04) |
| **Uterine cancer** | |  |  |  |  |  |  |
| Overall | | 1483.29(1317.51,1612.75) | 68.33(60.86,74.23) |  | 2329.07(2092.95,2560.89) | 53.54(48.13,58.84) | -0.22(-0.28, -0.14) |
| SDI regions | |  |  |  |  |  |  |
|  | High SDI | 380.07(361.66,395.57) | 66.52(63.07,69.26) |  | 596.79(555.85,636.05) | 65.31(60.99,69.65) | -0.02(-0.06, 0.03) |
|  | High-middle SDI | 523.24(486.39,558.71) | 86.43(80.17,92.33) |  | 667.94(606.21,735.23) | 61.50(55.81,67.85) | -0.29(-0.35, -0.22) |
|  | Middle SDI | 354.19(266.90,409.37) | 60.49(46.35,69.57) |  | 584.64(475.79,674.77) | 42.88(34.87,49.47) | -0.29(-0.40, -0.14) |
|  | Low-middle SDI | 159.43(129.51,193.56) | 49.01(39.98,59.48) |  | 332.22(280.87,404.90) | 44.53(37.69,54.57) | -0.09(-0.23, 0.07) |
|  | Low SDI | 65.37(51.43,82.35) | 51.50(40.84,65.01) |  | 145.26(117.90,181.52) | 51.24(41.73,64.02) | -0.01(-0.17, 0.23) |
| **Ovarian cancer** | |  |  |  |  |  |  |
| Overall | | 2732.67(2493.73,3165.17) | 124.09(113.68,142.97) |  | 5359.74(4692.95,5954.99) | 124.68(109.13,138.67) | 0.00(-0.15, 0.13) |
| SDI regions | |  |  |  |  |  |  |
|  | High SDI | 1061.10(956.79,1094.31) | 198.32(178.76,204.30) |  | 1229.12(1125.70,1323.42) | 143.78(132.56,154.51) | -0.28(-0.33, -0.14) |
|  | High-middle SDI | 864.81(793.57,925.11) | 145.06(132.93,155.31) |  | 1378.23(1191.05,1526.40) | 133.03(114.83,147.47) | -0.08(-0.20, 0.02) |
|  | Middle SDI | 462.01(401.88,592.52) | 73.86(64.70,93.48) |  | 1453.63(1199.32,1696.72) | 106.38(87.67,123.96) | 0.44(0.04, 0.73) |
|  | Low-middle SDI | 241.44(188.02,394.77) | 67.61(53.25,107.69) |  | 922.65(740.01,1169.38) | 118.41(95.38,150.23) | 0.75(0.07, 1.35) |
|  | Low SDI | 102.20(69.31,215.44) | 71.03(48.95,144.88) |  | 373.32(311.09,462.23) | 115.17(96.36,141.83) | 0.62(-0.06, 1.34) |

**Table S2.** The relative risks (95%CIs) of cervical cancer (CC) mortality attributable to unsafe sex due to age, period, and cohort effects, globally and each SDI regions separately

| **Value** | **Global** | **High SDI** | **High-middle SDI** | **Middle SDI** | **Low-middle SDI** | **Low SDI** |
| --- | --- | --- | --- | --- | --- | --- |
| **Age** |  |  |  |  |  |  |
| 15-19 | 0.02(0.00,0.32) | 0.01(0,2.06e+04) | 0.02(0.00,4.51) | 0.03(0.00,0.84) | 0.03(0.00,1.49) | 0.02(0.00,2.38) |
| 20-24 | 0.10(0.05,0.19) | 0.07(0.00,1.48) | 0.08(0.02,0.34) | 0.09(0.03,0.23) | 0.11(0.04,0.29) | 0.09(0.04,0.24) |
| 25-29 | 0.28(0.19,0.41) | 0.28(0.04,1.94) | 0.25(0.11,0.58) | 0.22(0.13,0.40) | 0.26(0.14,0.49) | 0.29(0.15,0.57) |
| 30-34 | 0.59(0.42,0.81) | 0.64(0.12,3.55) | 0.57(0.29,1.14) | 0.50(0.31,0.80) | 0.56(0.33,0.94) | 0.59(0.33,1.07) |
| 35-39 | 0.97(0.72,1.29) | 1.04(0.22,4.92) | 0.94(0.51,1.75) | 0.82(0.54,1.24) | 0.93(0.59,1.49) | 1.01(0.59,1.73) |
| 40-44 | 1.47(1.13,1.90) | 1.41(0.35,5.72) | 1.42(0.82,2.45) | 1.26(0.87,1.82) | 1.49(0.98,2.26) | 1.57(0.96,2.57) |
| 45-49 | 1.78(1.42,2.25) | 1.65(0.47,5.75) | 1.77(1.09,2.88) | 1.59(1.15,2.20) | 1.82(1.26,2.63) | 1.95(1.26,3.02) |
| 50-54 | 2.09(1.70,2.56) | 1.83(0.61,5.52) | 2.05(1.34,3.13) | 1.92(1.45,2.54) | 2.18(1.58,3.01) | 2.35(1.59,3.47) |
| 55-59 | 2.23(1.87,2.66) | 1.96(0.75,5.14) | 2.20(1.52,3.18) | 2.15(1.68,2.74) | 2.34(1.77,3.10) | 2.46(1.75,3.46) |
| 60-64 | 2.14(1.84,2.50) | 1.89(0.82,4.36) | 2.16(1.57,2.97) | 2.13(1.73,2.62) | 2.20(1.73,2.81) | 2.39(1.78,3.21) |
| 65-69 | 2.17(1.91,2.48) | 2.08(1.02,4.28) | 2.32(1.76,3.06) | 2.29(1.90,2.75) | 2.11(1.71,2.60) | 2.28(1.77,2.94) |
| 70-74 | 2.27(2.02,2.55) | 2.33(1.25,4.34) | 2.48(1.93,3.18) | 2.48(2.09,2.94) | 2.17(1.81,2.61) | 2.39(1.93,2.97) |
| 75-79 | 2.17(1.95,2.41) | 2.52(1.44,4.44) | 2.43(1.91,3.10) | 2.50(2.11,2.97) | 2.02(1.70,2.40) | 2.08(1.72,2.51) |
| 80-84 | 2.16(1.94,2.40) | 2.83(1.64,4.90) | 2.40(1.86,3.10) | 2.58(2.15,3.11) | 2.03(1.70,2.42) | 2.11(1.77,2.52) |
| 85-89 | 2.19(1.95,2.47) | 3.10(1.74,5.54) | 2.29(1.72,3.05) | 2.86(2.31,3.54) | 2.25(1.86,2.73) | 2.24(1.87,2.68) |
| 90-94 | 1.94(1.69,2.22) | 2.95(1.54,5.67) | 2.13(1.53,2.96) | 2.62(2.05,3.35) | 2.06(1.65,2.56) | 1.75(1.44,2.14) |
| **Period** |  |  |  |  |  |  |
| 1994 | 0.87(0.80,0.94) | 0.90(0.60,1.35) | 0.92(0.77,1.10) | 0.86(0.76,0.98) | 0.88(0.77,1.00) | 0.86(0.75,0.98) |
| 1999 | 0.91(0.87,0.96) | 0.94(0.74,1.21) | 0.93(0.83,1.03) | 0.91(0.84,0.98) | 0.93(0.86,1.01) | 0.91(0.83,0.98) |
| 2004 | 0.98(0.97,1.00) | 0.97(0.90,1.06) | 0.98(0.94,1.01) | 1.01(0.99,1.04) | 0.98(0.95,1.01) | 0.98(0.95,1.01) |
| 2009 | 1.01(1.00,1.03) | 0.99(0.91,1.08) | 1.00(0.96,1.04) | 1.04(1.01,1.06) | 1.01(0.98,1.04) | 1.03(1.00,1.06) |
| 2014 | 1.08(1.03,1.13) | 1.05(0.82,1.35) | 1.06(0.95,1.18) | 1.08(1.00,1.17) | 1.07(0.99,1.15) | 1.09(1.01,1.19) |
| 2019 | 1.17(1.08,1.26) | 1.16(0.77,1.75) | 1.14(0.95,1.37) | 1.13(0.99,1.28) | 1.16(1.02,1.32) | 1.17(1.02,1.35) |
| **Cohort** |  |  |  |  |  |  |
| 1904-1908 | 2.52(1.72,3.68) | 2.80(0.35,22.25) | 2.63(1.20,5.79) | 2.77(1.61,4.77) | 2.33(1.21,4.50) | 2.35(1.10,5.02) |
| 1909-1913 | 2.43(1.68,3.53) | 2.69(0.35,20.80) | 2.57(1.19,5.54) | 2.72(1.61,4.61) | 2.33(1.22,4.45) | 2.31(1.09,4.90) |
| 1914-1918 | 2.25(1.56,3.25) | 2.54(0.34,19.30) | 2.46(1.16,5.22) | 2.41(1.44,4.03) | 2.24(1.18,4.24) | 2.27(1.07,4.79) |
| 1919-1923 | 2.10(1.46,3.03) | 2.29(0.30,17.30) | 2.28(1.08,4.80) | 2.12(1.28,3.54) | 2.15(1.14,4.06) | 2.17(1.03,4.58) |
| 1924-1928 | 1.91(1.33,2.76) | 2.17(0.28,16.52) | 2.05(0.97,4.31) | 1.99(1.19,3.30) | 1.99(1.05,3.78) | 2.01(0.95,4.27) |
| 1929-1933 | 1.72(1.19,2.49) | 1.90(0.24,14.79) | 1.80(0.85,3.82) | 1.79(1.07,2.99) | 1.78(0.93,3.40) | 1.84(0.86,3.93) |
| 1934-1938 | 1.56(1.07,2.27) | 1.69(0.21,13.61) | 1.58(0.73,3.38) | 1.62(0.96,2.73) | 1.58(0.82,3.04) | 1.67(0.77,3.62) |
| 1939-1943 | 1.40(0.96,2.06) | 1.50(0.18,12.70) | 1.39(0.64,3.05) | 1.46(0.86,2.49) | 1.40(0.71,2.73) | 1.50(0.68,3.31) |
| 1944-1948 | 1.26(0.85,1.87) | 1.29(0.14,11.59) | 1.22(0.55,2.75) | 1.31(0.75,2.28) | 1.27(0.64,2.53) | 1.37(0.61,3.07) |
| 1949-1953 | 1.12(0.75,1.69) | 1.14(0.12,10.95) | 1.10(0.47,2.54) | 1.18(0.67,2.10) | 1.14(0.56,2.33) | 1.23(0.54,2.83) |
| 1954-1958 | 1.02(0.67,1.56) | 1.03(0.10,10.75) | 1.03(0.43,2.46) | 1.06(0.58,1.94) | 1.03(0.49,2.14) | 1.10(0.47,2.60) |
| 1959-1963 | 0.93(0.60,1.45) | 0.93(0.08,10.58) | 0.93(0.37,2.32) | 0.95(0.51,1.79) | 0.94(0.44,2.02) | 1.00(0.41,2.43) |
| 1964-1968 | 0.85(0.53,1.34) | 0.82(0.07,10.27) | 0.83(0.32,2.17) | 0.87(0.45,1.68) | 0.86(0.39,1.90) | 0.88(0.35,2.21) |
| 1969-1973 | 0.75(0.47,1.22) | 0.72(0.05,9.92) | 0.73(0.27,1.99) | 0.76(0.38,1.53) | 0.77(0.34,1.74) | 0.76(0.29,1.97) |
| 1974-1978 | 0.67(0.40,1.11) | 0.63(0.04,9.61) | 0.64(0.22,1.85) | 0.66(0.32,1.37) | 0.67(0.28,1.57) | 0.67(0.25,1.80) |
| 1979-1983 | 0.62(0.37,1.05) | 0.55(0.03,9.47) | 0.59(0.20,1.80) | 0.59(0.27,1.27) | 0.62(0.25,1.51) | 0.60(0.21,1.66) |
| 1984-1988 | 0.55(0.31,0.95) | 0.48(0.02,9.35) | 0.54(0.17,1.75) | 0.50(0.22,1.14) | 0.56(0.22,1.42) | 0.51(0.18,1.49) |
| 1989-1993 | 0.47(0.26,0.86) | 0.43(0.02,9.91) | 0.46(0.13,1.63) | 0.42(0.17,1.04) | 0.49(0.18,1.34) | 0.44(0.14,1.36) |
| 1994-1998 | 0.42(0.20,0.89) | 0.39(0.01,13.09) | 0.38(0.07,1.97) | 0.36(0.11,1.26) | 0.42(0.12,1.50) | 0.38(0.11,1.35) |
| 1999-2003 | 0.38(0.08,1.80) | 0.32(0.00,775.05) | 0.36(0.01,15.8) | 0.33(0.02,5.28) | 0.37(0.03,4.46) | 0.33(0.03,3.58) |
| 2004-2008 | 0.32(0,1074.51) | 0.29(0.00,1.23e+19) | 0.32(0.00,4.99e+06) | 0.30(0.00,2.25e+04) | 0.3(0.00,4.00e+05 | 0.28(0.00,5.05e+06 |

**Table S3.** The relative risks (95%CIs) of cervical cancer (CC) mortality attributable to smoking due to age, period, and cohort effects, globally and each SDI regions separately

| **Value** | **Global** | **High SDI** | **High-middle SDI** | **Middle SDI** | **Low-middle SDI** | **Low SDI** |
| --- | --- | --- | --- | --- | --- | --- |
| **Age** |  |  |  |  |  |  |
| 30-34 | 0.15(0.12,0.21) | 0.22(0.17,0.28) | 0.21(0.18,0.25) | 0.11(0.06,0.21) | 0.11(0.06,0.23) | 0.11(0.08,0.17) |
| 35-39 | 0.32(0.28,0.36) | 0.43(0.38,0.49) | 0.40(0.37,0.44) | 0.24(0.18,0.32) | 0.24(0.17,0.32) | 0.27(0.23,0.32) |
| 40-44 | 0.59(0.54,0.64) | 0.69(0.63,0.75) | 0.69(0.66,0.73) | 0.47(0.39,0.56) | 0.51(0.42,0.62) | 0.56(0.51,0.61) |
| 45-49 | 0.86(0.81,0.92) | 0.93(0.86,0.99) | 1.02(0.97,1.06) | 0.69(0.60,0.80) | 0.78(0.67,0.90) | 0.93(0.86,1.00) |
| 50-54 | 1.18(1.12,1.24) | 1.11(1.05,1.18) | 1.29(1.24,1.34) | 1.02(0.91,1.15) | 1.24(1.10,1.39) | 1.32(1.25,1.41) |
| 55-59 | 1.47(1.41,1.54) | 1.31(1.25,1.38) | 1.53(1.48,1.58) | 1.40(1.27,1.54) | 1.59(1.44,1.75) | 1.69(1.61,1.78) |
| 60-64 | 1.48(1.42,1.54) | 1.28(1.22,1.34) | 1.43(1.39,1.47) | 1.52(1.41,1.64) | 1.58(1.45,1.71) | 1.81(1.74,1.89) |
| 65-69 | 1.58(1.53,1.63) | 1.43(1.38,1.49) | 1.48(1.45,1.52) | 1.74(1.63,1.86) | 1.64(1.54,1.76) | 1.57(1.51,1.63) |
| 70-74 | 1.70(1.65,1.75) | 1.50(1.45,1.55) | 1.52(1.49,1.56) | 2.00(1.89,2.13) | 1.79(1.69,1.91) | 1.64(1.59,1.70) |
| 75-79 | 1.46(1.41,1.51) | 1.40(1.35,1.45) | 1.28(1.25,1.31) | 1.76(1.65,1.88) | 1.43(1.34,1.53) | 1.17(1.13,1.22) |
| 80-84 | 1.54(1.49,1.60) | 1.39(1.33,1.45) | 1.28(1.24,1.31) | 1.94(1.80,2.10) | 1.75(1.62,1.88) | 1.63(1.58,1.69) |
| 85-89 | 1.68(1.61,1.75) | 1.51(1.45,1.58) | 1.33(1.29,1.36) | 2.23(2.02,2.45) | 2.06(1.89,2.25) | 1.89(1.82,1.97) |
| 90-94 | 1.53(1.46,1.61) | 1.43(1.36,1.51) | 1.21(1.17,1.25) | 2.04(1.82,2.29) | 2.00(1.80,2.22) | 1.66(1.58,1.74) |
| **Period** |  |  |  |  |  |  |
| 1994 | 1.1(1.07,1.13) | 1.16(1.12,1.19) | 1.07(1.05,1.09) | 1.10(1.04,1.18) | 1.05(0.99,1.11) | 1.00(0.97,1.03) |
| 1999 | 1.08(1.05,1.1) | 1.09(1.06,1.12) | 1.04(1.02,1.05) | 1.11(1.07,1.15) | 1.05(1.01,1.09) | 1.01(0.99,1.03) |
| 2004 | 1.06(1.05,1.08) | 1.01(0.99,1.04) | 1.06(1.05,1.08) | 1.13(1.10,1.15) | 1.09(1.07,1.11) | 1.03(1.02,1.05) |
| 2009 | 0.97(0.96,0.99) | 0.95(0.92,0.97) | 1.00(0.98,1.01) | 0.98(0.96,1.00) | 1.01(0.99,1.03) | 1.00(0.98,1.01) |
| 2014 | 0.92(0.9,0.94) | 0.92(0.89,0.94) | 0.94(0.93,0.96) | 0.89(0.85,0.93) | 0.93(0.89,0.97) | 0.98(0.96,1.00) |
| 2019 | 0.89(0.86,0.92) | 0.90(0.87,0.93) | 0.90(0.88,0.92) | 0.84(0.78,0.89) | 0.89(0.84,0.95) | 0.98(0.95,1.01) |
| **Cohort** |  |  |  |  |  |  |
| 1904-1908 | 1.55(1.43,1.67) | 1.38(1.27,1.50) | 1.32(1.24,1.40) | 2.00(1.67,2.41) | 1.61(1.35,1.91) | 1.51(1.38,1.65) |
| 1909-1913 | 1.57(1.46,1.68) | 1.39(1.30,1.50) | 1.39(1.32,1.45) | 2.01(1.69,2.38) | 1.71(1.46,2.00) | 1.56(1.44,1.69) |
| 1914-1918 | 1.49(1.40,1.60) | 1.37(1.29,1.46) | 1.38(1.32,1.44) | 1.76(1.49,2.07) | 1.67(1.44,1.94) | 1.56(1.45,1.69) |
| 1919-1923 | 1.45(1.36,1.55) | 1.33(1.25,1.41) | 1.36(1.31,1.42) | 1.54(1.31,1.81) | 1.65(1.42,1.91) | 1.48(1.38,1.60) |
| 1924-1928 | 1.39(1.30,1.48) | 1.34(1.26,1.42) | 1.30(1.26,1.36) | 1.45(1.24,1.70) | 1.52(1.32,1.76) | 1.38(1.28,1.49) |
| 1929-1933 | 1.27(1.19,1.36) | 1.23(1.16,1.31) | 1.17(1.13,1.22) | 1.32(1.12,1.55) | 1.39(1.20,1.61) | 1.32(1.22,1.42) |
| 1934-1938 | 1.20(1.12,1.28) | 1.14(1.07,1.22) | 1.14(1.09,1.18) | 1.21(1.02,1.43) | 1.24(1.06,1.45) | 1.26(1.17,1.37) |
| 1939-1943 | 1.12(1.04,1.20) | 1.07(1.00,1.15) | 1.05(1.01,1.10) | 1.13(0.94,1.35) | 1.13(0.96,1.33) | 1.20(1.10,1.30) |
| 1944-1948 | 1.07(0.99,1.16) | 1.04(0.97,1.13) | 1.04(1.00,1.09) | 1.05(0.86,1.27) | 1.06(0.89,1.26) | 1.13(1.04,1.24) |
| 1949-1953 | 1.02(0.94,1.11) | 1.02(0.94,1.11) | 1.02(0.97,1.08) | 0.97(0.79,1.19) | 0.98(0.81,1.19) | 1.07(0.97,1.18) |
| 1954-1958 | 0.98(0.89,1.08) | 1.01(0.92,1.10) | 1.02(0.97,1.08) | 0.90(0.72,1.12) | 0.91(0.74,1.12) | 0.99(0.89,1.09) |
| 1959-1963 | 0.95(0.86,1.04) | 1.00(0.91,1.11) | 0.99(0.93,1.05) | 0.85(0.67,1.08) | 0.87(0.70,1.09) | 0.92(0.83,1.03) |
| 1964-1968 | 0.87(0.78,0.97) | 0.96(0.86,1.06) | 0.88(0.83,0.94) | 0.81(0.62,1.05) | 0.82(0.64,1.05) | 0.84(0.75,0.95) |
| 1969-1973 | 0.75(0.66,0.85) | 0.85(0.75,0.96) | 0.76(0.70,0.82) | 0.71(0.53,0.96) | 0.71(0.54,0.94) | 0.74(0.65,0.85) |
| 1974-1978 | 0.66(0.56,0.78) | 0.73(0.63,0.86) | 0.72(0.65,0.79) | 0.62(0.43,0.89) | 0.60(0.42,0.85) | 0.63(0.54,0.74) |
| 1979-1983 | 0.62(0.49,0.77) | 0.65(0.51,0.81) | 0.72(0.63,0.83) | 0.56(0.33,0.95) | 0.56(0.34,0.92) | 0.57(0.46,0.72) |
| 1984-1988 | 0.57(0.38,0.86) | 0.58(0.39,0.87) | 0.70(0.55,0.87) | 0.49(0.17,1.40) | 0.55(0.21,1.42) | 0.53(0.34,0.83) |
| 1989-1993 | 0.52(0.17,1.64) | 0.58(0.22,1.57) | 0.61(0.33,1.14) | 0.47(0.03,8.60) | 0.50(0.04,7.23) | 0.50(0.13,1.95) |

**Table S4.** The relative risks (95%CIs) of uterine cancer (UC) mortality attributable to high body mass index (BMI) due to age, period, and cohort effects, globally and each SDI regions separately

| **Value** | **Global** | **High SDI** | **High-middle SDI** | **Middle SDI** | **Low-middle SDI** | **Low SDI** |
| --- | --- | --- | --- | --- | --- | --- |
| **Age** |  |  |  |  |  |  |
| 20-24 | 0.02(0,3.37e+03) | 0.01(0,4.61e+10) | 0.02(0,6.92e+04) | 0.04(0.00,0.64) | 0.03(0.00,25.82) | 0.02(0.0,141.07) |
| 25-29 | 0.06(0.00,2.99) | 0.04(0,323.49) | 0.07(0.00,3.82) | 0.09(0.03,0.25) | 0.08(0.01,0.62) | 0.07(0.01,0.69) |
| 30-34 | 0.13(0.01,1.31) | 0.12(0.00,16.01) | 0.15(0.01,1.58) | 0.18(0.10,0.33) | 0.15(0.04,0.55) | 0.15(0.03,0.64) |
| 35-39 | 0.27(0.04,1.61) | 0.25(0.00,14.79) | 0.30(0.05,1.93) | 0.34(0.21,0.54) | 0.29(0.11,0.81) | 0.27(0.08,0.93) |
| 40-44 | 0.52(0.11,2.44) | 0.47(0.01,17.26) | 0.55(0.11,2.77) | 0.63(0.42,0.94) | 0.60(0.25,1.44) | 0.65(0.22,1.89) |
| 45-49 | 0.96(0.25,3.72) | 0.88(0.04,21.32) | 1.01(0.25,4.13) | 1.14(0.81,1.61) | 1.09(0.50,2.37) | 1.12(0.43,2.91) |
| 50-54 | 1.53(0.47,4.95) | 1.54(0.10,24.90) | 1.58(0.46,5.36) | 1.67(1.24,2.25) | 1.73(0.88,3.39) | 1.89(0.82,4.37) |
| 55-59 | 2.22(0.81,6.08) | 2.46(0.22,27.04) | 2.30(0.81,6.54) | 2.16(1.68,2.78) | 2.37(1.33,4.24) | 2.78(1.34,5.75) |
| 60-64 | 3.23(1.38,7.57) | 3.91(0.51,29.94) | 3.23(1.33,7.86) | 2.87(2.33,3.54) | 3.40(2.08,5.56) | 4.13(2.22,7.68) |
| 65-69 | 3.87(1.89,7.92) | 4.76(0.86,26.27) | 3.89(1.82,8.29) | 3.15(2.64,3.75) | 3.80(2.52,5.74) | 4.47(2.65,7.54) |
| 70-74 | 4.10(2.22,7.59) | 5.15(1.22,21.77) | 4.02(2.05,7.87) | 3.23(2.78,3.76) | 3.70(2.62,5.21) | 4.02(2.60,6.21) |
| 75-79 | 4.01(2.27,7.09) | 5.02(1.41,17.91) | 3.76(1.97,7.19) | 3.02(2.62,3.49) | 3.49(2.58,4.71) | 3.96(2.74,5.71) |
| 80-84 | 3.78(2.10,6.82) | 4.54(1.31,15.73) | 3.43(1.71,6.88) | 2.72(2.33,3.16) | 2.97(2.23,3.97) | 2.95(2.12,4.11) |
| 85-89 | 3.90(1.99,7.62) | 4.44(1.14,17.28) | 3.24(1.46,7.20) | 2.81(2.36,3.36) | 2.91(2.12,3.99) | 2.57(1.83,3.61) |
| 90-94 | 4.10(1.85,9.07) | 4.14(0.84,20.35) | 3.44(1.35,8.81) | 2.98(2.41,3.69) | 2.95(2.03,4.28) | 2.33(1.59,3.43) |
| **Period** |  |  |  |  |  |  |
| 1994 | 0.67(0.41,1.10) | 0.57(0.19,1.71) | 0.81(0.47,1.38) | 0.62(0.55,0.71) | 0.54(0.41,0.70) | 0.53(0.39,0.71) |
| 1999 | 0.80(0.59,1.07) | 0.74(0.38,1.43) | 0.85(0.62,1.17) | 0.77(0.72,0.84) | 0.70(0.60,0.82) | 0.67(0.56,0.80) |
| 2004 | 0.95(0.86,1.04) | 0.93(0.75,1.16) | 0.93(0.83,1.03) | 0.97(0.94,0.99) | 0.94(0.89,0.99) | 0.91(0.86,0.97) |
| 2009 | 1.09(0.99,1.21) | 1.12(0.90,1.40) | 1.03(0.93,1.15) | 1.15(1.12,1.18) | 1.15(1.09,1.22) | 1.14(1.07,1.21) |
| 2014 | 1.26(0.94,1.69) | 1.37(0.71,2.63) | 1.17(0.85,1.62) | 1.29(1.19,1.39) | 1.42(1.21,1.66) | 1.46(1.22,1.75) |
| 2019 | 1.43(0.87,2.35) | 1.64(0.55,4.90) | 1.30(0.76,2.22) | 1.45(1.27,1.65) | 1.74(1.34,2.27) | 1.88(1.39,2.54) |
| **Cohort** |  |  |  |  |  |  |
| 1904-1908 | 3.92(0.79,19.44) | 5.44(0.14,209.06) | 2.81(0.45,17.55) | 2.97(2.03,4.35) | 3.67(1.63,8.27) | 3.81(1.45,10.01) |
| 1909-1913 | 3.60(0.80,16.33) | 4.63(0.15,147.44) | 2.83(0.50,16.17) | 2.76(1.94,3.93) | 3.21(1.50,6.86) | 3.36(1.35,8.35) |
| 1914-1918 | 3.26(0.77,13.77) | 3.85(0.14,106.66) | 2.86(0.53,15.24) | 2.48(1.78,3.44) | 2.78(1.35,5.72) | 2.98(1.25,7.12) |
| 1919-1923 | 2.66(0.66,10.78) | 3.10(0.12,78.54) | 2.58(0.50,13.20) | 2.14(1.57,2.93) | 2.49(1.24,4.98) | 2.55(1.09,5.95) |
| 1924-1928 | 2.43(0.61,9.64) | 2.62(0.11,64.65) | 2.44(0.48,12.30) | 1.99(1.47,2.70) | 2.16(1.09,4.27) | 2.15(0.93,4.99) |
| 1929-1933 | 2.03(0.50,8.14) | 2.04(0.08,51.85) | 2.25(0.44,11.50) | 1.78(1.31,2.41) | 1.80(0.91,3.59) | 1.83(0.78,4.29) |
| 1934-1938 | 1.66(0.40,6.95) | 1.64(0.06,45.44) | 1.93(0.36,10.30) | 1.58(1.15,2.17) | 1.53(0.75,3.10) | 1.60(0.66,3.84) |
| 1939-1943 | 1.45(0.32,6.44) | 1.37(0.04,43.78) | 1.75(0.31,9.99) | 1.42(1.02,1.98) | 1.33(0.63,2.79) | 1.39(0.55,3.50) |
| 1944-1948 | 1.23(0.25,5.95) | 1.17(0.03,45.15) | 1.45(0.23,9.04) | 1.28(0.89,1.83) | 1.18(0.53,2.61) | 1.23(0.46,3.27) |
| 1949-1953 | 1.08(0.20,5.81) | 1.03(0.02,49.96) | 1.21(0.17,8.46) | 1.15(0.78,1.70) | 1.07(0.45,2.51) | 1.11(0.39,3.14) |
| 1954-1958 | 0.95(0.16,5.78) | 0.89(0.01,55.99) | 1.07(0.14,8.49) | 1.03(0.67,1.57) | 0.97(0.38,2.43) | 0.96(0.31,2.93) |
| 1959-1963 | 0.84(0.12,5.81) | 0.77(0.01,65.10) | 0.91(0.10,8.30) | 0.92(0.58,1.45) | 0.87(0.32,2.36) | 0.85(0.25,2.82) |
| 1964-1968 | 0.69(0.09,5.54) | 0.63(0.01,72.86) | 0.72(0.07,7.65) | 0.79(0.48,1.30) | 0.75(0.26,2.19) | 0.73(0.20,2.66) |
| 1969-1973 | 0.56(0.06,5.27) | 0.52(0.00,83.76) | 0.55(0.04,6.89) | 0.65(0.38,1.12) | 0.63(0.20,2.02) | 0.63(0.16,2.50) |
| 1974-1978 | 0.48(0.04,5.27) | 0.43(0.00,99.38) | 0.46(0.03,6.89) | 0.55(0.30,0.99) | 0.54(0.15,1.87) | 0.54(0.12,2.38) |
| 1979-1983 | 0.43(0.03,5.79) | 0.39(0.00,131.69) | 0.42(0.02,7.81) | 0.49(0.26,0.94) | 0.49(0.13,1.88) | 0.47(0.09,2.31) |
| 1984-1988 | 0.37(0.02,7.22) | 0.34(0.00,205.06) | 0.37(0.01,10.16) | 0.42(0.20,0.91) | 0.41(0.09,1.94) | 0.40(0.07,2.35) |
| 1989-1993 | 0.32(0.01,19.81) | 0.32(0,>100.00) | 0.30(0.00,34.30) | 0.35(0.11,1.09) | 0.36(0.04,2.88) | 0.34(0.04,3.12) |
| 1994-1998 | 0.28(0,1.20e+03) | 0.26(0,1.14e+07) | 0.26(0,5.40e+04) | 0.33(0.04,2.88) | 0.31(0.01,14.99) | 0.29(0.00,18.85) |
| 1999-2003 | 0.25(0,2.36e+12) | 0.21(0,4.69e+29) | 0.24(0,2.67e+14) | 0.31(0,238.21) | 0.26(0,1.02e+06) | 0.24(0,4.05e+07) |

**Table S5.** The relative risks of ovarian cancer (OC) mortality attributable to high BMI due to age, period, and cohort effects, globally and each SDI regions separately

| **Value** | **Global** | **High SDI** | **High-middle SDI** | **Middle SDI** | **Low-middle SDI** | **Low SDI** |
| --- | --- | --- | --- | --- | --- | --- |
| **Age** |  |  |  |  |  |  |
| 20-24 | 0.04(0.00,0.77) | 0.05(0.00,0.61) | 0.04(0.00,2.30) | 0.08(0.03,0.19) | 0.06(0.02,0.21) | 0.06(0.02,0.21) |
| 25-29 | 0.08(0.02,0.32) | 0.09(0.02,0.31) | 0.08(0.01,0.51) | 0.14(0.09,0.21) | 0.12(0.07,0.21) | 0.12(0.07,0.20) |
| 30-34 | 0.16(0.07,0.36) | 0.16(0.07,0.33) | 0.17(0.06,0.48) | 0.24(0.18,0.31) | 0.22(0.15,0.30) | 0.22(0.16,0.30) |
| 35-39 | 0.31(0.17,0.55) | 0.28(0.17,0.48) | 0.34(0.16,0.72) | 0.40(0.33,0.49) | 0.39(0.30,0.50) | 0.38(0.30,0.48) |
| 40-44 | 0.63(0.39,1.01) | 0.56(0.37,0.86) | 0.73(0.39,1.34) | 0.73(0.62,0.86) | 0.74(0.61,0.92) | 0.76(0.62,0.92) |
| 45-49 | 1.20(0.80,1.79) | 1.05(0.74,1.50) | 1.38(0.81,2.35) | 1.27(1.11,1.46) | 1.32(1.11,1.58) | 1.42(1.20,1.68) |
| 50-54 | 1.89(1.34,2.66) | 1.69(1.26,2.28) | 2.10(1.34,3.30) | 1.89(1.68,2.12) | 2.06(1.77,2.40) | 2.30(1.99,2.66) |
| 55-59 | 2.35(1.77,3.14) | 2.20(1.71,2.81) | 2.59(1.77,3.78) | 2.17(1.97,2.39) | 2.32(2.04,2.64) | 2.58(2.28,2.91) |
| 60-64 | 2.77(2.19,3.50) | 2.70(2.21,3.31) | 2.95(2.16,4.03) | 2.42(2.23,2.62) | 2.62(2.35,2.91) | 3.02(2.73,3.34) |
| 65-69 | 3.09(2.55,3.73) | 3.11(2.64,3.67) | 3.21(2.49,4.15) | 2.54(2.38,2.70) | 2.77(2.55,3.02) | 2.92(2.70,3.17) |
| 70-74 | 3.20(2.74,3.74) | 3.36(2.90,3.89) | 3.14(2.53,3.89) | 2.54(2.41,2.68) | 2.75(2.57,2.94) | 2.86(2.68,3.05) |
| 75-79 | 3.08(2.67,3.55) | 3.33(2.87,3.87) | 2.81(2.29,3.45) | 2.32(2.21,2.43) | 2.54(2.40,2.68) | 2.55(2.42,2.69) |
| 80-84 | 2.71(2.32,3.16) | 2.99(2.51,3.56) | 2.25(1.79,2.82) | 1.89(1.80,1.99) | 1.99(1.88,2.10) | 1.78(1.69,1.88) |
| 85-89 | 2.86(2.37,3.45) | 3.07(2.48,3.80) | 2.19(1.67,2.88) | 2.04(1.91,2.17) | 2.01(1.88,2.15) | 1.76(1.65,1.88) |
| 90-94 | 2.68(2.12,3.39) | 2.72(2.10,3.53) | 2.08(1.49,2.91) | 1.92(1.78,2.08) | 1.73(1.59,1.88) | 1.31(1.20,1.42) |
| **Period** |  |  |  |  |  |  |
| 1994 | 0.64(0.55,0.75) | 0.72(0.62,0.84) | 0.69(0.56,0.85) | 0.52(0.49,0.54) | 0.41(0.39,0.44) | 0.46(0.43,0.49) |
| 1999 | 0.78(0.71,0.86) | 0.85(0.78,0.93) | 0.79(0.70,0.90) | 0.69(0.67,0.71) | 0.61(0.59,0.64) | 0.61(0.58,0.63) |
| 2004 | 0.94(0.91,0.98) | 0.96(0.93,0.99) | 0.95(0.91,0.99) | 0.91(0.90,0.92) | 0.91(0.90,0.93) | 0.88(0.86,0.89) |
| 2009 | 1.12(1.09,1.16) | 1.08(1.05,1.11) | 1.10(1.06,1.15) | 1.17(1.15,1.18) | 1.22(1.20,1.24) | 1.17(1.15,1.19) |
| 2014 | 1.28(1.17,1.41) | 1.18(1.08,1.29) | 1.24(1.09,1.41) | 1.47(1.42,1.52) | 1.65(1.58,1.71) | 1.60(1.54,1.66) |
| 2019 | 1.48(1.26,1.72) | 1.32(1.14,1.53) | 1.40(1.14,1.73) | 1.80(1.71,1.90) | 2.16(2.02,2.31) | 2.19(2.06,2.33) |
| **Cohort** |  |  |  |  |  |  |
| 1904-1908 | 3.41(2.31,5.04) | 2.97(1.97,4.48) | 2.45(1.43,4.18) | 2.38(2.09,2.72) | 2.88(2.45,3.38) | 2.61(2.23,3.06) |
| 1909-1913 | 3.11(2.21,4.39) | 2.76(1.91,4.01) | 2.52(1.56,4.06) | 2.31(2.07,2.59) | 2.54(2.21,2.91) | 2.40(2.10,2.74) |
| 1914-1918 | 2.79(2.06,3.78) | 2.55(1.82,3.58) | 2.48(1.62,3.79) | 2.02(1.83,2.22) | 2.21(1.97,2.48) | 2.25(2.01,2.53) |
| 1919-1923 | 2.33(1.77,3.06) | 2.37(1.73,3.25) | 2.15(1.46,3.17) | 1.77(1.63,1.93) | 1.96(1.77,2.17) | 1.95(1.77,2.16) |
| 1924-1928 | 2.12(1.65,2.73) | 2.20(1.62,2.97) | 2.03(1.41,2.92) | 1.64(1.52,1.77) | 1.70(1.56,1.86) | 1.67(1.53,1.83) |
| 1929-1933 | 1.75(1.36,2.24) | 1.89(1.40,2.55) | 1.85(1.29,2.65) | 1.48(1.38,1.59) | 1.45(1.33,1.58) | 1.47(1.34,1.60) |
| 1934-1938 | 1.44(1.11,1.87) | 1.64(1.21,2.24) | 1.63(1.12,2.37) | 1.35(1.26,1.46) | 1.27(1.16,1.38) | 1.32(1.21,1.44) |
| 1939-1943 | 1.26(0.95,1.67) | 1.47(1.06,2.04) | 1.48(0.99,2.22) | 1.26(1.16,1.37) | 1.14(1.03,1.26) | 1.20(1.08,1.32) |
| 1944-1948 | 1.08(0.79,1.48) | 1.28(0.90,1.83) | 1.26(0.80,1.97) | 1.18(1.07,1.30) | 1.05(0.93,1.18) | 1.10(0.98,1.23) |
| 1949-1953 | 0.95(0.66,1.35) | 1.12(0.76,1.66) | 1.07(0.65,1.77) | 1.08(0.96,1.21) | 0.98(0.85,1.12) | 1.02(0.89,1.16) |
| 1954-1958 | 0.83(0.55,1.24) | 0.95(0.62,1.47) | 0.96(0.54,1.69) | 0.97(0.86,1.11) | 0.92(0.78,1.07) | 0.93(0.80,1.08) |
| 1959-1963 | 0.75(0.47,1.17) | 0.82(0.51,1.33) | 0.85(0.45,1.59) | 0.91(0.79,1.05) | 0.86(0.72,1.02) | 0.86(0.72,1.02) |
| 1964-1968 | 0.64(0.39,1.07) | 0.69(0.41,1.18) | 0.71(0.35,1.43) | 0.82(0.69,0.96) | 0.77(0.63,0.94) | 0.78(0.65,0.95) |
| 1969-1973 | 0.55(0.31,0.97) | 0.58(0.33,1.04) | 0.57(0.26,1.23) | 0.70(0.59,0.85) | 0.68(0.55,0.86) | 0.71(0.57,0.88) |
| 1974-1978 | 0.50(0.27,0.94) | 0.50(0.26,0.95) | 0.51(0.22,1.20) | 0.64(0.52,0.78) | 0.62(0.48,0.80) | 0.65(0.51,0.82) |
| 1979-1983 | 0.49(0.24,0.99) | 0.46(0.22,0.95) | 0.49(0.19,1.28) | 0.60(0.48,0.76) | 0.59(0.44,0.78) | 0.60(0.46,0.78) |
| 1984-1988 | 0.47(0.20,1.11) | 0.43(0.17,1.08) | 0.46(0.14,1.47) | 0.54(0.41,0.72) | 0.55(0.39,0.76) | 0.55(0.40,0.75) |
| 1989-1993 | 0.44(0.12,1.56) | 0.40(0.10,1.58) | 0.41(0.07,2.38) | 0.46(0.30,0.71) | 0.50(0.31,0.79) | 0.49(0.32,0.77) |
| 1994-1998 | 0.44(0.04,4.29) | 0.36(0.03,4.63) | 0.40(0.01,11.42) | 0.45(0.22,0.92) | 0.47(0.21,1.05) | 0.45(0.20,0.98) |
| 1999-2003 | 0.44(0,115.09) | 0.31(0,181.21) | 0.42(0,1.25e+03) | 0.44(0.08,2.28) | 0.45(0.06,3.44) | 0.41(0.05,3.06) |

**Table S6.** The relative risks of ovarian cancer (OC) mortality attributable to occupational asbestos exposure due to age, period, and cohort effects, globally and each SDI regions separately

| **Value** | **Global** | **High SDI** | **High-middle SDI** | **Middle SDI** | **Low-middle SDI** | **Low SDI** |
| --- | --- | --- | --- | --- | --- | --- |
| **Age** |  |  |  |  |  |  |
| 20-24 | 0.01(0.00,>100) | 0.00(0.00,>100) | 0.01(0.00,>100) | 0.01(0.00,>100) | 0.01(0.00,>100) | 0.01(0.00,>100) |
| 25-29 | 0.02(0.00,>100) | 0.01(0.00,>100) | 0.03(0.00,>100) | 0.04(0.00,>100) | 0.03(0.00,>100) | 0.02(0.00,>100) |
| 30-34 | 0.02(0.00,>100) | 0.00(0.00,>100) | 0.02(0.00,>100) | 0.04(0.00,>100) | 0.04(0.00,>100) | 0.03(0.00,>100) |
| 35-39 | 0.12(0.00,>100) | 0.05(0.00,>100) | 0.19(0.00,>100) | 0.13(0.00,>100) | 0.14(0.00,>100) | 0.16(0.00,>100) |
| 40-44 | 0.31(0.00,>100) | 0.26(0.00,>100) | 0.38(0.00,>100) | 0.37(0.00,>100) | 0.46(0.00,>100) | 0.56(0.00,>100) |
| 45-49 | 0.55(0.00,>100) | 0.71(0.00,>100) | 0.69(0.00,>100) | 0.53(0.00,>100) | 0.67(0.00,>100) | 0.82(0.00,>100) |
| 50-54 | 1.38(0.00,>100) | 2.04(0.00,>100) | 1.59(0.00,>100) | 1.16(0.00,>100) | 1.71(0.00,>100) | 2.19(0.00,>100) |
| 55-59 | 2.67(0.00,>100) | 4.33(0.00,>100) | 2.92(0.00,>100) | 2.23(0.00,>100) | 2.87(0.00,>100) | 3.54(0.00,>100) |
| 60-64 | 4.06(0.00,>100) | 7.55(0.00,>100) | 4.21(0.00,>100) | 2.98(0.00,>100) | 3.39(0.01,>100) | 4.05(0.00,>100) |
| 65-69 | 6.16(0.00,>100) | 11.80(0.00,>100) | 5.86(0.00,>100) | 4.36(0.01, >100) | 4.78(0.03,>100) | 5.33(0.00,>100) |
| 70-74 | 8.70(0.00,>100) | 16.40(0.00,>100) | 7.45(0.00,>100) | 6.56(0.01, >100) | 6.72(0.09,>100) | 7.44(0.00,>100) |
| 75-79 | 11.06(0.00,>100) | 20.27(0.00,>100) | 8.56(0.00,>100) | 7.81(0.00,>100) | 7.71(0.14,>100) | 8.05(0.00,>100) |
| 80-84 | 12.84(0.00,>100) | 21.94(0.00,>100) | 8.95(0.00,>100) | 9.11(0.00,>100) | 8.54(0.12,>100) | 8.46(0.00,>100) |
| 85-89 | 13.89(0.00,>100) | 21.18(0.00,>100) | 9.25(0.00,>100) | 10.67(0.00,>100) | 8.16(0.06,>100) | 7.09(0.00,>100) |
| 90-94 | 11.68(0.00,>100) | 15.42(0.00,>100) | 8.12(0.00,>100) | 10.30(0.00,>100) | 7.16(0.02,>100) | 5.78(0.00,>100) |
| **Period** |  |  |  |  |  |  |
| 1994 | 0.65(0.00,>100) | 0.63(0.00,>100) | 0.69(0.00,>100) | 0.60(0,169.49) | 0.48(0.01,19.06) | 0.52(0.00,>100) |
| 1999 | 0.76(0.00,>100) | 0.74(0.00,>100) | 0.80(0.00,>100) | 0.73(0.02,21.46) | 0.64(0.07,5.84) | 0.67(0.02,23.42) |
| 2004 | 0.89(0.12,6.45) | 0.86(0.04,17.59) | 0.93(0.11,8.26) | 0.90(0.29,2.79) | 0.88(0.42,1.84) | 0.86(0.26,2.83) |
| 2009 | 1.11(0.15,8.09) | 1.09(0.05,22.29) | 1.12(0.13,9.89) | 1.14(0.37,3.54) | 1.16(0.55,2.41) | 1.11(0.34,3.64) |
| 2014 | 1.32(0.00,>100) | 1.36(0.00,>100) | 1.25(0.00,>100) | 1.38(0.05,40.75) | 1.55(0.17,14.09) | 1.48(0.04,52.15) |
| 2019 | 1.54(0.00,>100) | 1.67(0.00,>100) | 1.40(0.00,>100) | 1.62(0.01,>100) | 2.05(0.05,81.09) | 2.04(0.01,>100) |
| **Cohort** |  |  |  |  |  |  |
| 1904-1908 | 7.26(0.00,>100) | 11.25(0.00,>100) | 4.85(0.00,>100) | 5.11(0.00,>100) | 6.61(0.00,>100) | 5.68(0.00,>100) |
| 1909-1913 | 6.65(0.00,>100) | 9.94(0.00,>100) | 4.97(0.00,>100) | 4.91(0.00,>100) | 5.74(0.00,>100) | 5.13(0.00,>100) |
| 1914-1918 | 6.03(0.00,>100) | 8.70(0.00,>100) | 5.09(0.00,>100) | 4.17(0.00,>100) | 4.55(0.00,>100) | 4.76(0.00,>100) |
| 1919-1923 | 4.86(0.00,>100) | 7.41(0.00,>100) | 4.16(0.00,>100) | 3.41(0.00,>100) | 3.85(0.00,>100) | 4.03(0.00,>100) |
| 1924-1928 | 4.32(0.00,>100) | 6.41(0.00,>100) | 3.75(0.00,>100) | 3.02(0.00,>100) | 3.10(0.00,>100) | 3.30(0.00,>100) |
| 1929-1933 | 3.25(0.00,>100) | 4.86(0.00,>100) | 3.05(0.00,>100) | 2.58(0.00,>100) | 2.49(0.00,>100) | 2.77(0.00,>100) |
| 1934-1938 | 2.51(0.00,>100) | 3.78(0.00,>100) | 2.59(0.00,>100) | 2.19(0.00,>100) | 2.03(0.00,>100) | 2.38(0.00,>100) |
| 1939-1943 | 2.03(0.00,>100) | 2.97(0.00,>100) | 2.23(0.00,>100) | 1.94(0.00,>100) | 1.72(0.00,>100) | 2.03(0.00,>100) |
| 1944-1948 | 1.62(0.00,>100) | 2.21(0.00,>100) | 1.86(0.00,>100) | 1.68(0.00,>100) | 1.42(0.00,>100) | 1.69(0.00,>100) |
| 1949-1953 | 1.28(0.00,>100) | 1.64(0.00,>100) | 1.43(0.00,>100) | 1.45(0.00,>100) | 1.21(0.00,>100) | 1.40(0.00,>100) |
| 1954-1958 | 0.94(0.00,>100) | 1.12(0.00,>100) | 1.11(0.00,>100) | 1.22(0.00,>100) | 1.00(0.00,>100) | 1.14(0.00,>100) |
| 1959-1963 | 0.74(0.00,>100) | 0.79(0.00,>100) | 0.89(0.00,>100) | 1.04(0.00,>100) | 0.83(0.00,>100) | 0.91(0.00,>100) |
| 1964-1968 | 0.58(0.00,>100) | 0.57(0.00,>100) | 0.68(0.00,>100) | 0.85(0.00,>100) | 0.68(0.00,>100) | 0.71(0.00,>100) |
| 1969-1973 | 0.45(0.00,>100) | 0.40(0.00,>100) | 0.49(0.00,>100) | 0.65(0.00,>100) | 0.56(0.00,>100) | 0.55(0.00,>100) |
| 1974-1978 | 0.34(0.00,>100) | 0.27(0.00,>100) | 0.37(0.00,>100) | 0.48(0.00,>100) | 0.41(0.00,>100) | 0.42(0.00,>100) |
| 1979-1983 | 0.29(0.00,>100) | 0.20(0.00,>100) | 0.30(0.00,>100) | 0.40(0.00,>100) | 0.35(0.00,>100) | 0.32(0.00,>100) |
| 1984-1988 | 0.23(0.00,>100) | 0.13(0.00,>100) | 0.26(0.00,>100) | 0.28(0.00,>100) | 0.29(0.00,>100) | 0.24(0.00,>100) |
| 1989-1993 | 0.12(0.00,>100) | 0.05(0.00,>100) | 0.11(0.00,>100) | 0.11(0.00,>100) | 0.18(0.00,>100) | 0.15(0.00,>100) |
| 1994-1998 | 0.10(0.00,>100) | 0.07(0.00,>100) | 0.11(0.00,>100) | 0.09(0.00,>100) | 0.15(0.00,>100) | 0.11(0.00,>100) |
| 1999-2003 | 0.09(0.00,>100) | 0.02(0.00,>100) | 0.09(0.00,>100) | 0.09(0.00,>100) | 0.10(0.00,>100) | 0.08(0.00,>100) |

**Table S7.** The relative risks of ovarian cancer (OC) mortality attributable to high fasting plasma glucose (FPG) due to age, period, and cohort effects, globally and each SDI regions separately

| **Value** | **Global** | | | **High SDI** | **High-middle SDI** | | **Middle SDI** | | **Low-middle SDI** | | **Low SDI** | |
| --- | --- | --- | --- | --- | --- | --- | --- | --- | --- | --- | --- | --- |
| **Age** | |  |  | | |  | |  | |  | |  |
| 25-29 | | 0.02(0.00,23.69) | 0.02(0,>100.00) | | | 0.02(0,>100.00) | | 0.03(0.00,5.50) | | 0.03(0.00,0.96) | | 0.03(0.00,0.45) |
| 30-34 | | 0.05(0.00,0.82) | 0.05(0.00,5.40) | | | 0.06(0.00,2.97) | | 0.07(0.01,0.54) | | 0.06(0.02,0.25) | | 0.06(0.02,0.18) |
| 35-39 | | 0.12(0.02,0.54) | 0.10(0.01,1.45) | | | 0.13(0.01,1.16) | | 0.14(0.04,0.46) | | 0.14(0.06,0.31) | | 0.14(0.08,0.26) |
| 40-44 | | 0.29(0.09,0.93) | 0.25(0.03,1.86) | | | 0.32(0.06,1.72) | | 0.32(0.13,0.78) | | 0.35(0.19,0.63) | | 0.35(0.22,0.56) |
| 45-49 | | 0.69(0.25,1.86) | 0.62(0.11,3.43) | | | 0.74(0.17,3.17) | | 0.74(0.34,1.57) | | 0.77(0.46,1.29) | | 0.79(0.53,1.17) |
| 50-54 | | 1.37(0.58,3.24) | 1.27(0.29,5.57) | | | 1.43(0.41,5.02) | | 1.42(0.74,2.73) | | 1.55(1.00,2.42) | | 1.61(1.14,2.27) |
| 55-59 | | 2.06(0.99,4.31) | 2.08(0.59,7.33) | | | 2.16(0.74,6.29) | | 2.08(1.19,3.63) | | 2.10(1.44,3.07) | | 2.19(1.63,2.94) |
| 60-64 | | 2.91(1.57,5.38) | 3.06(1.06,8.79) | | | 2.98(1.22,7.29) | | 2.86(1.80,4.54) | | 2.87(2.10,3.94) | | 3.03(2.37,3.87) |
| 65-69 | | 3.66(2.21,6.06) | 3.98(1.66,9.52) | | | 3.71(1.77,7.78) | | 3.46(2.37,5.06) | | 3.47(2.68,4.49) | | 3.56(2.91,4.35) |
| 70-74 | | 4.26(2.82,6.43) | 4.71(2.29,9.73) | | | 4.17(2.25,7.75) | | 3.94(2.89,5.38) | | 3.91(3.18,4.80) | | 4.05(3.45,4.76) |
| 75-79 | | 4.42(3.10,6.29) | 5.00(2.64,9.46) | | | 4.17(2.40,7.25) | | 3.85(2.94,5.05) | | 3.94(3.33,4.67) | | 4.12(3.61,4.70) |
| 80-84 | | 4.33(3.07,6.12) | 4.97(2.63,9.40) | | | 3.84(2.19,6.73) | | 3.59(2.74,4.71) | | 3.68(3.15,4.30) | | 3.57(3.16,4.03) |
| 85-89 | | 4.22(2.86,6.24) | 4.68(2.27,9.66) | | | 3.63(1.91,6.89) | | 3.45(2.52,4.71) | | 3.33(2.81,3.95) | | 3.18(2.78,3.63) |
| 90-94 | | 3.45(2.15,5.56) | 3.68(1.54,8.79) | | | 3.04(1.40,6.58) | | 2.71(1.85,3.96) | | 2.30(1.87,2.84) | | 1.97(1.67,2.32) |
| **Period** | |  |  | | |  | |  | |  | |  |
| 1994 | | 0.53(0.38,0.76) | 0.59(0.32,1.08) | | | 0.55(0.33,0.92) | | 0.51(0.39,0.66) | | 0.44(0.37,0.52) | | 0.46(0.40,0.52) |
| 1999 | | 0.68(0.55,0.84) | 0.71(0.49,1.02) | | | 0.70(0.51,0.96) | | 0.67(0.57,0.79) | | 0.61(0.55,0.68) | | 0.62(0.57,0.67) |
| 2004 | | 0.91(0.85,0.98) | 0.88(0.78,1.00) | | | 1.00(0.90,1.11) | | 0.91(0.86,0.96) | | 0.88(0.85,0.91) | | 0.87(0.85,0.90) |
| 2009 | | 1.20(1.12,1.29) | 1.17(1.03,1.32) | | | 1.22(1.10,1.36) | | 1.18(1.11,1.25) | | 1.19(1.15,1.23) | | 1.18(1.15,1.21) |
| 2014 | | 1.43(1.16,1.77) | 1.39(0.96,1.99) | | | 1.35(0.99,1.84) | | 1.49(1.27,1.75) | | 1.62(1.46,1.80) | | 1.60(1.48,1.74) |
| 2019 | | 1.76(1.24,2.49) | 1.69(0.92,3.10) | | | 1.58(0.94,2.66) | | 1.85(1.41,2.43) | | 2.19(1.84,2.61) | | 2.15(1.88,2.46) |
| **Cohort** | |  |  | | |  | |  | |  | |  |
| 1904-1908 | | 4.29(1.76,10.5) | 4.1(0.74,22.57) | | | 3.56(0.82,15.41) | | 3.74(1.85,7.54) | | 4.04(2.67,6.10) | | 3.79(2.74,5.24) |
| 1909-1913 | | 3.83(1.71,8.57) | 3.72(0.77,17.96) | | | 3.40(0.88,13.13) | | 3.53(1.87,6.64) | | 3.37(2.35,4.84) | | 3.29(2.47,4.39) |
| 1914-1918 | | 3.31(1.59,6.87) | 3.31(0.76,14.40) | | | 3.14(0.89,11.10) | | 2.86(1.60,5.08) | | 2.77(2.01,3.81) | | 2.82(2.19,3.65) |
| 1919-1923 | | 2.67(1.36,5.27) | 2.88(0.71,11.68) | | | 2.59(0.78,8.64) | | 2.32(1.35,3.96) | | 2.33(1.75,3.11) | | 2.34(1.86,2.96) |
| 1924-1928 | | 2.32(1.21,4.47) | 2.55(0.65,10.05) | | | 2.26(0.70,7.35) | | 2.00(1.19,3.35) | | 1.96(1.50,2.57) | | 1.95(1.57,2.44) |
| 1929-1933 | | 1.83(0.95,3.53) | 2.08(0.52,8.29) | | | 1.85(0.56,6.07) | | 1.70(1.01,2.86) | | 1.65(1.26,2.16) | | 1.67(1.34,2.08) |
| 1934-1938 | | 1.49(0.75,2.97) | 1.72(0.41,7.23) | | | 1.60(0.46,5.52) | | 1.46(0.84,2.51) | | 1.40(1.05,1.87) | | 1.44(1.14,1.82) |
| 1939-1943 | | 1.26(0.59,2.66) | 1.48(0.32,6.81) | | | 1.36(0.36,5.06) | | 1.27(0.71,2.30) | | 1.22(0.88,1.67) | | 1.27(0.98,1.64) |
| 1944-1948 | | 1.08(0.47,2.46) | 1.22(0.23,6.39) | | | 1.18(0.28,4.89) | | 1.12(0.58,2.14) | | 1.07(0.75,1.54) | | 1.11(0.83,1.48) |
| 1949-1953 | | 0.92(0.37,2.30) | 1.02(0.17,6.15) | | | 1.00(0.21,4.69) | | 0.97(0.47,1.99) | | 0.96(0.64,1.45) | | 1.00(0.72,1.38) |
| 1954-1958 | | 0.77(0.28,2.15) | 0.83(0.12,5.94) | | | 0.84(0.15,4.56) | | 0.84(0.38,1.86) | | 0.85(0.54,1.36) | | 0.87(0.60,1.26) |
| 1959-1963 | | 0.67(0.22,2.09) | 0.69(0.08,5.92) | | | 0.72(0.11,4.58) | | 0.75(0.31,1.81) | | 0.76(0.45,1.28) | | 0.76(0.50,1.15) |
| 1964-1968 | | 0.57(0.16,2.01) | 0.56(0.05,5.85) | | | 0.61(0.08,4.57) | | 0.65(0.24,1.73) | | 0.65(0.36,1.17) | | 0.65(0.41,1.03) |
| 1969-1973 | | 0.49(0.12,1.97) | 0.46(0.04,5.90) | | | 0.51(0.06,4.54) | | 0.56(0.19,1.64) | | 0.56(0.29,1.07) | | 0.56(0.34,0.94) |
| 1974-1978 | | 0.44(0.10,1.99) | 0.40(0.02,6.32) | | | 0.44(0.04,4.78) | | 0.49(0.15,1.61) | | 0.48(0.23,0.98) | | 0.49(0.28,0.86) |
| 1979-1983 | | 0.40(0.07,2.24) | 0.36(0.02,8.23) | | | 0.40(0.03,5.87) | | 0.45(0.12,1.70) | | 0.43(0.19,0.97) | | 0.42(0.22,0.80) |
| 1984-1988 | | 0.37(0.03,3.88) | 0.32(0.00,24.57) | | | 0.36(0.01,14.93) | | 0.39(0.06,2.49) | | 0.39(0.13,1.15) | | 0.37(0.16,0.88) |
| 1989-1993 | | 0.32(0.00,39.99) | 0.27(0,2.03e+03) | | | 0.31(0,8.01e+02) | | 0.31(0.01,14.61) | | 0.34(0.04,2.96) | | 0.33(0.06,1.79) |
| 1994-1998 | | 0.29(0,3.15e+05) | 0.22(0,4.41e+11) | | | 0.27(0,9.86e+09) | | 0.28(0,1.43e+04) | | 0.31(0.00,121.91) | | 0.29(0.00,37.46) |
